# *Prionochelys matutina* Zangerl, 1953 (Testudines: Pan-Cheloniidae) from the Late Cretaceous of the United States and the evolution of epithecal ossifications in marine turtles

**DOI:** 10.7717/peerj.5876

**Published:** 2018-11-01

**Authors:** Andrew D. Gentry

**Affiliations:** Department of Biological Sciences, University of Alabama at Birmingham, Birmingham, AL, United States of America; McWane Science Center, Birmingham, AL, United States of America

**Keywords:** Western Interior Seaway, Marine turtle, Mooreville Chalk, Mississippi Embayment, Fossil, Campanian

## Abstract

**Background:**

Many neritic to nearshore species of marine adapted turtle from the Late Cretaceous of North America are thought to represent the stem lineage of Cheloniidae but due to fragmentary holotypes, low total specimen counts, and resultantly incomplete morphological character suites, are routinely placed either within or outside of crown group Chelonioidea leaving their precise cladistic affinities uncertain. Despite this systematic ambiguity, the referral of these species to either the stem of Cheloniidae or Chelonioidea belies the critical importance of these taxa in any investigation into the origins of extant marine turtles. The adequate incorporation of these species into phylogenetic studies requires the formal description of relatively complete specimens, particularly those possessing associated cranial and post-cranial material.

**Methods:**

Remarkably complete fossil specimens of several adult and juvenile marine turtles from the Mooreville Chalk and Eutaw Formations (Alabama, USA) are formally described and assigned to *Prionochelys matutina*. This material provides new information into the anatomy, ontogeny, and cladistic affinities of the species. A phylogenetic hypothesis for Late Cretaceous marine turtles is then generated through the consilience of stratigraphic, morphological, and molecular data.

**Results:**

Phylogenetic analysis places *Prionochelys matutina* on the stem of Cheloniidae as a member of a monophyletic clade with other putative pan-cheloniids, including *Ctenochelys stenoporus*, *Ctenochelys acris*, *Peritresius martini*, and *Peritresius ornatus*. The members of this clade possess incipient secondary palates, pronounced carapacial and plastral fontanelles at all stages of development, and are characterized by the presence of superficial ossifications at the apices of the neural keel elevations along the dorsal midline of the carapace.

**Discussion:**

The epithecal osteoderms dorsal to the neural series (epineurals) found in Ctenochelyidae are unique among turtles. The presence of epineurals in ctenochelyid turtles shows that epithecal ossifications arose independently in both leatherback (Dermochelyidae) and hard-shelled (Cheloniidae) marine turtles. Whether or not the epineurals of Ctenochelyidae are homologous with the dermal ossicles comprising the carapace of *Dermochelys coriacea* remains untested however, histological thin sectioning of dermochelyid and ctenochelyd epithecal elements may reveal meaningful information in future studies.

## Introduction

Of all the genera of fossil marine adapted turtle recovered from the Late Cretaceous of North America, perhaps one of the poorest known is *Prionochelys* Zangerl, 1953. *Prionochelys* or the ‘saw-tooth turtle’ is a spectacularly ornamented sea turtle characterized by pronounced peripheral serrations and an undulating sagittal keel along the dorsal midline of the carapace. As a result of the largely incomplete holotype specimens for the various species of *Prionochelys* and the limited amount of historically referred material, the precise morphology of *Prionochelys* has been essentially speculative despite the frequent usage of *Prionochelys* reconstructions in natural history museum exhibits. The lack of information available for this genus has also lead to confusion regarding the taxonomy of *Prionochelys*. Zangerl (1953) initially described three species of *Prionochelys* based on minor differences in neural arrangements and the geographic location each species was discovered: *P. matutina* Zangerl, 1953 from the Mooreville Chalk of Alabama, *P. nauta* Zangerl, 1953 from the Marlbrook Marl of Arkansas, and *P. galeotergum* Zangerl, 1953 from the Niobrara Formation of Kansas. Nearly 50 years later, Hirayama (1997) postulated that *P. galeotergum* and *P. matutina* were junior synonyms of *P. nauta* but did not provide any justification for this systematic revision. This assessment is not supported here. Hirayama also hypothesized that *Prionochelys* was closely related to *Peritresius* Leidy, 1856 and *Ctenochelys* Zangerl, 1953 based on the presence of epithecal ossifications dorsal to the neuralia (epineurals). Until now, this evolutionary relationship has never been explicitly tested within a phylogenetic context owing primarily to the lack of available anatomical information for species of *Prionochelys.*

One of the purposes of this study is to describe newly identified material belonging to *Prionochelys* from the collections at McWane Science Center in Birmingham, Alabama, USA and the Alabama Museum of Natural History in Tuscaloosa, Alabama, USA. The cranial material associated with MSC 39030 represents the most complete skull yet identified for any species of *Prionochelys* and allows for the first detailed description and comparison of *Prionochelys* cranial elements with those of other closely related Cretaceous marine turtles. MSC 3500 is only the second definitively juvenile individual of *Prionochelys* to be identified and by far the most complete juvenile known. This specimen provides the opportunity to examine ontogenetic variation within the genus. ALMNH 6673 is the first material of *Prionochelys nauta* discovered outside of Arkansas and is the oldest occurrence of the species. Collectively, the specimens referenced herein help to elucidate the morphology of *Prionochelys* thereby allowing for the first comprehensive investigation into the taxonomy and cladistic affinities of this poorly known member of Pan-Cheloniidae (sensu Joyce, Parham & Gauthier, 2004).

## Material and Methods

### Localities and geological setting

**Figure 1:**
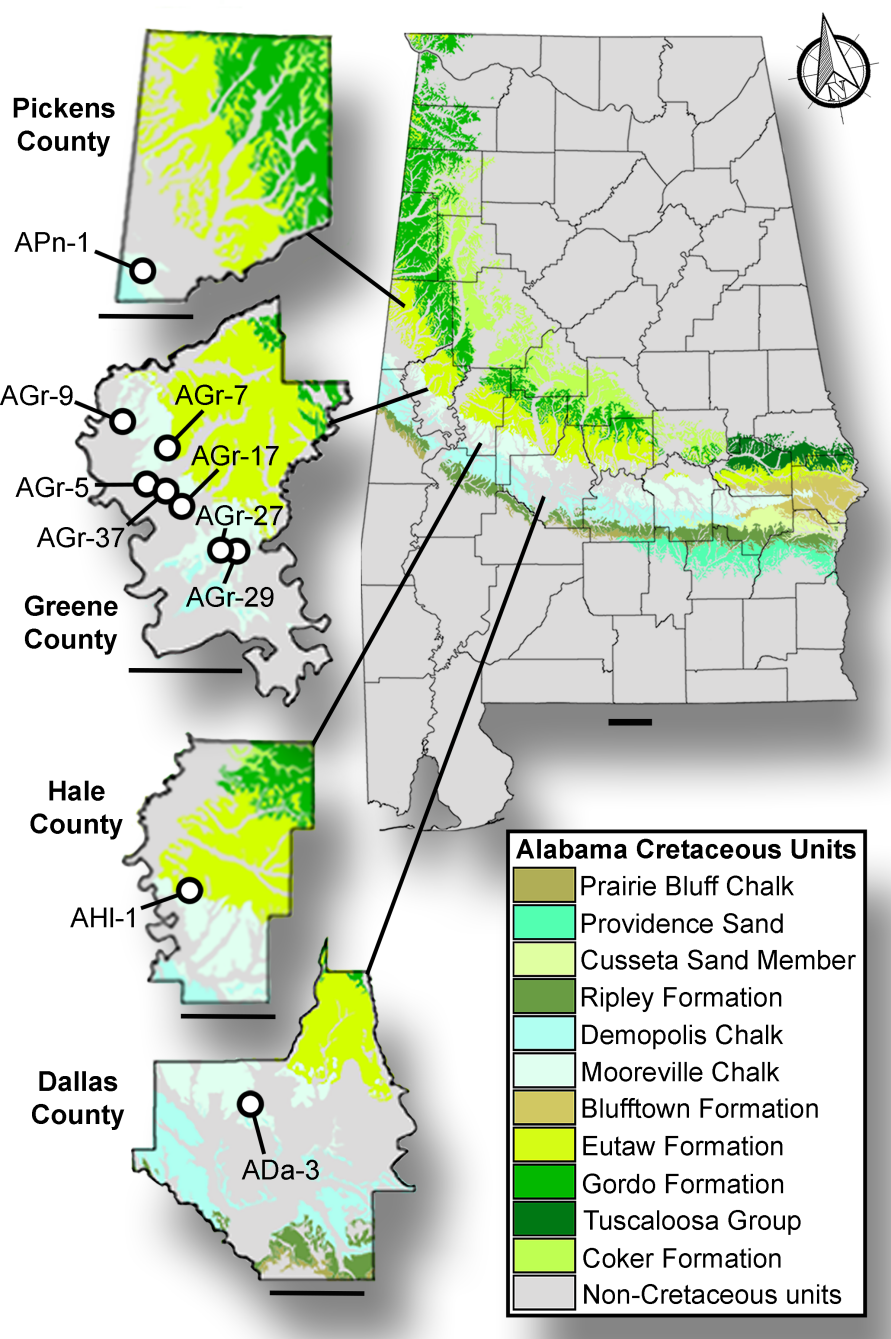
Surface stratigraphy of Alabama *Prionochelys matutina* localities. Upper Cretaceous surface exposures of Alabama and the localization of discussed *Prionochelys matutina* specimens. Scale bars = 30 km.

**Figure 2:**
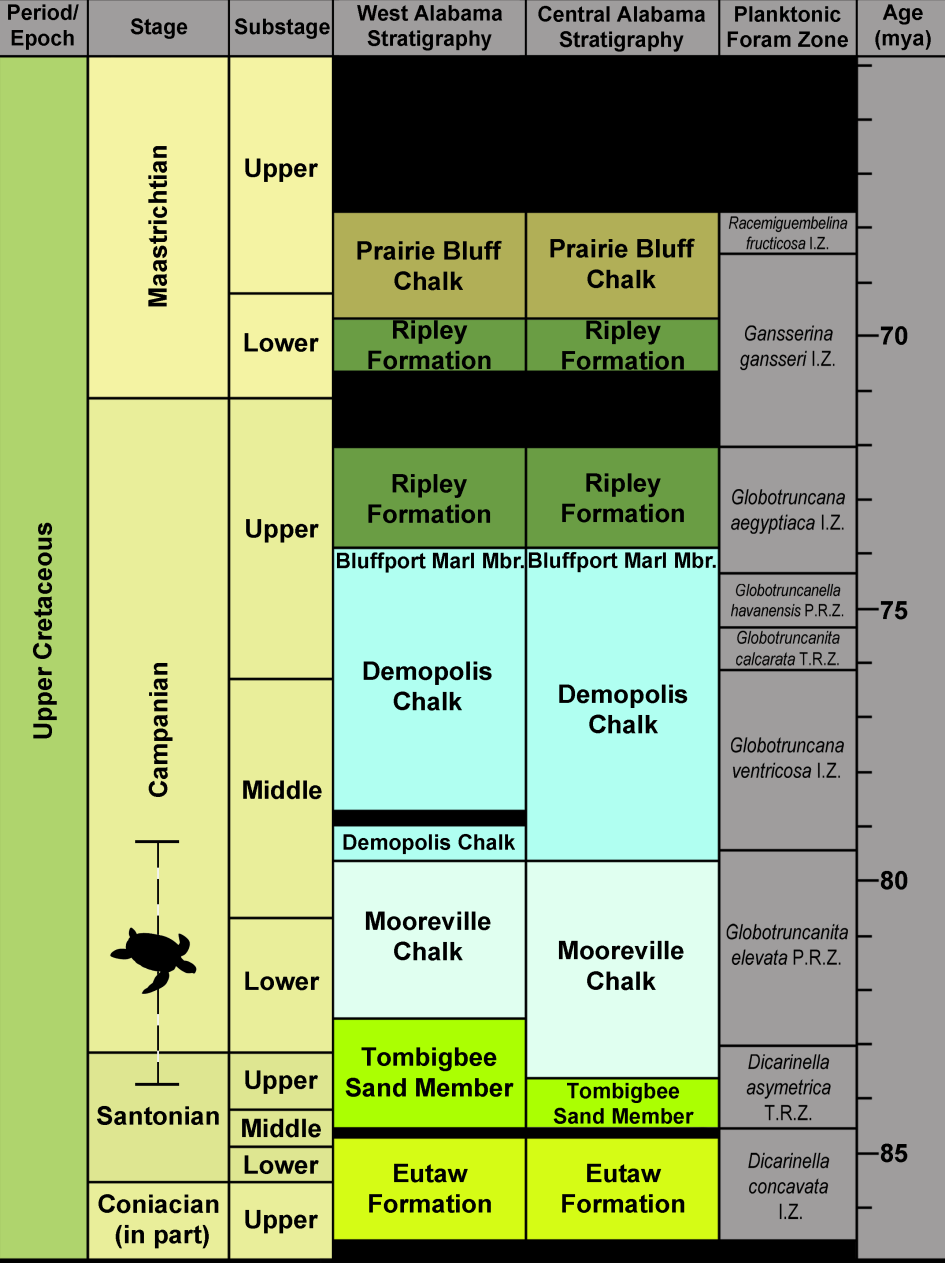
Generalized Santonian through Campanian surface stratigraphy of western and central Alabama. Stratigraphy follows that of Mancini, Puckett & Tew (1996) and Dockery (2008). Planktonic foraminferal zones after Caron (1985) and Mancini & Puckett (2005). Modified from Gentry et al. (2018).

The *Prionochelys matutina* specimens identified in this study were collected from multiple sites from two upper Cretaceous formations in Alabama, USA (Fig. 1, Table 1). The majority of the specimens were recovered from the Upper Cretaceous Mooreville Chalk which spans the upper Santonian in the central part of Alabama to middle Campanian at the western edge of the state (Fig. 2; Puckett, 1994; Mancini, Puckett & Tew, 1996; Liu, 2007). The Mooreville Chalk has been interpreted to represent a middle neritic to nearshore environment (Raymond et al., 1988; Puckett, 1992). Only a single specimen (AHl-1) was recovered from the middle Santonian to lower Campanian Tombigbee Sand Member of the Eutaw Formation. The Tombigbee Sand Member is interpreted as being a middle neritic environment based on the presence of numerous burrows and mollusk shells (Raymond et al., 1988; Puckett, 2005).

Within this study, all localities are referenced by standard Alabama and Mississippi site file numbers. All cited localities are located on private property; however, permission was obtained by MSC to collect at these locations. Precise locality information for each specimen, though not provided here, is on file at MSC and fully available to qualified researchers.

### Material

The *Prionochelys* specimens described in this study are from the collections housed at the McWane Science Center (MSC) in Birmingham, Alabama, USA and the Alabama Museum of Natural History (ALMNH) in Tuscaloosa, Alabama, USA (Table S1). Specimens were prepared and cleaned using manual preparation techniques and water. When necessary, broken elements were repaired using B-76 butvar. The specimens were photographed using a Nikon D3300 camera and images were processed using Adobe Photoshop version 2014 software as part of the production of the figures. Osteological terminology pertaining to the skull primarily follows that of Gaffney (1972), but includes recent adjustments to the terminology concerning the pathways and foramina of the carotid arteries (Rabi et al., 2013). Postcranial osteological terminology follows Zangerl (1953).

**Table 1:** List of relevant stratigraphic units, localities, and specimens. Lithology and depositional descriptions follow Raymond et al. (1988) and Puckett (1992).

**Age**	**Unit**	**Lithology**	**Depositional setting**	**Locality**	**County**	**State**	**Specimens**
Campanian	Mooreville Chalk	Compact fossiliferous clayey chalk and chalky marl	Inner to middle neritic environments below the wave base	AGr-37	Greene	AL	*P. matutina*: MSC 3500
				AGr-29	Greene	AL	*P. matutina*: MSC 2720
				AGr-27	Greene	AL	*P. matutina*: MSC 2610
				AGr-17	Greene	AL	*P. matutina*: MSC 6086
				AGr-9	Greene	AL	*P. matutina*: MSC 5626
				AGr-7	Greene	AL	*P. matutina*: MSC 39013, MSC 3036, MSC 1719
				AGr-5	Greene	AL	*P. matutina*: MSC 1915, MSC 2045,MSC 39030
				ADa-3	Greene	AL	*P. matutina*: MSC 38604, MSC 3140
				APn-1	Pickens	AL	*P. matutina*: MSC 1540
Sant.	Eutaw Formation-Tombigbee Sand Member	Loosely compacted, fossiliferous sandy clay	Inner to middle neritic environments below the wave base	AHl-1	Hale	AL	*P. matutina*: MSC 2250

### Phylogenetic methods

The cladistic affinities of *P. matutina* were tested within a phylogenetic framework using a modified version of the Cadena & Parham (2015) matrix (Files S1 & S2). The matrix consists of 257 characters scored for 28 species and was altered to include recent character adjustments for *Toxochelys latiremis* Cope, 1873, *Ctenochelys stenoporus* (Hay, 1905), and *Ctenochelys acris* Zangerl, 1953 (Gentry, 2016). Scorings for *Peritresius* spp. come from Gentry et al. (2018). One new binary state character was added regarding the presence/absence of epineural ossifications (ch. 127), an apomorphy of at least three genera of Cretaceous pan-cheloniid (*Ctenochelys* Zangerl, 1953, *Peritresius* Leidy, 1856, and *Prionochelys*). Parsimony analyses were performed using the software PAUP* version 4.0a (build 163) and the heuristic search algorithm. This process was followed by Tree Bisection Reconnection (TBR) branch swapping (saving 10 trees per replication). Trees were constrained using a molecular constraint tree of extant taxa (Crawford et al., 2014; File S3). Support for each node was calculated using the bootstrap resampling method. The 37 ordered characters from the Cadena & Parham (2015) matrix were also used in the present study. Terminal operational taxonomic units (OTUs) were limited to individual species. Phylogenetic nomenclature and definitions follow Joyce (2007) and Joyce et al. (2013). Numbers in parentheses refer to characters used in the phylogenetic analyses and their corresponding scores.

## New Specimens of *Prionochelys Matutina*

**Figure 3:**
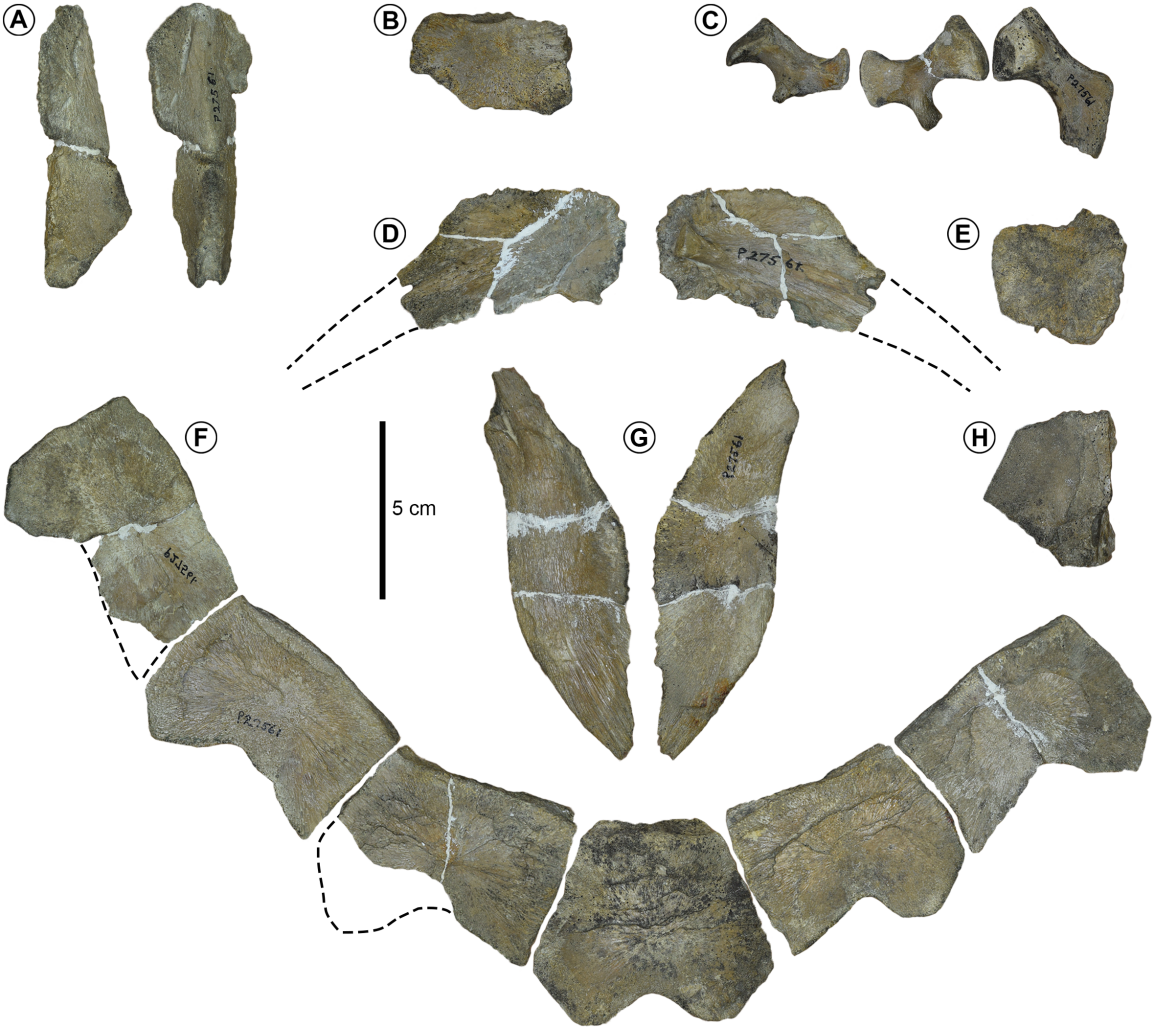
Holotype of *Prionochelys matutina* (P27561) from the Mooreville Chalk of Alabama. (A) Neurals in lateral view on the left and dorsal view on the right. (B) Neural fragment in lateral view. (C) Left ischium, right ischium, and right ilium in ventral view. (D) Posterior costal (7?) in dorsal view on the left and ventral view on the right. (E) Costal fragment in dorsal view. (F) Posterior peripherals and pygal in dorsal view. (G) Left xiphiplastron in dorsal view on the left and ventral view on the right. (H) Peripheral fragment in dorsal(?) view.

**Holotype** FMNH P27561, Fig. 3; Zangerl, 1953, fig. 118, p. 255.

**Referred material** MSC 3500, MSC 6086, MSC 3036, MSC 2720, MSC 1915, MSC 39030, MSC 39013, MSC 38604, MSC 2610, MSC 2045, MSC 3140, MSC 1719, MSC 5626.

**Horizon and locality** Greene County, Alabama. Mooreville Chalk Formation, Lower Campanian. Pickens County, Alabama. Mooreville Chalk Formation, Lower Campanian. Hale County, Alabama. Eutaw Formation, Tombigbee Sand Member, Middle Santonian-Lower Campanian.

### Description

#### Skull

**Figure 4:**
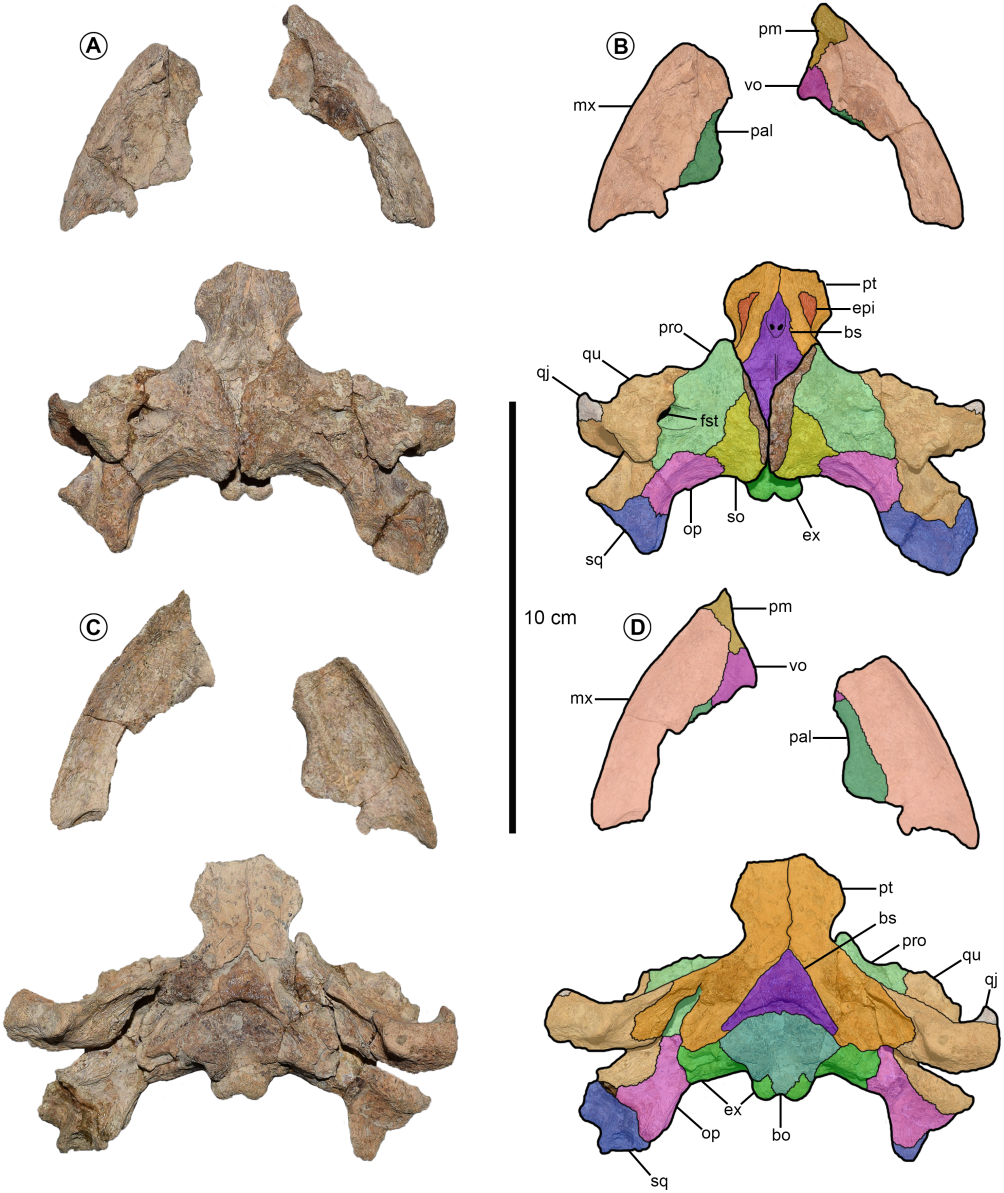
*Prionochelys matutina* (MSC 39030) from the Mooreville Chalk of Alabama. (A, C) Preserved cranial material in dorsal view on top and ventral view below. (B, D) Illustrations of cranial material in dorsal view on top and ventral view below. Abbreviations: bo, basioccipital; bs, basisphenoid; epi, epipterygoids; ex, exoccipital; fst, foramen stapedio-temporale; mx, maxilla; op, opisthotic; pal, palatine; pm, premaxilla; pro, prootic; pt, pterygoid; qj, quadratojugal; qu, quadrate; so, supraoccipital; sq, squamosal; vo, vomer.

The skull associated with MSC 39030 (Fig. 4) is the first known cranial material of *Prionochelys matutina*. The supraoccipital crest and the anterior portions of the pterygoids are missing so it is impossible to determine an exact length for the skull but it is estimated at approximately 12 cm. The width of the skull is roughly 9.5 cm. The skull has a broadly rounded anterior margin though not as broad as that of *Ctenochelys acris* (Gentry, 2016; RMM 6157, fig. 4, p. 6) and not as pointed as *Ctenochelys stenoporus* (Zangerl, 1953) or *Toxochelys latiremis* (Matzke, 2009; USNM 18252, text-fig. 1, p. 99). The skull roof is missing from MSC 39030 and these elements remain unknown for the species.

**Figure 5:**
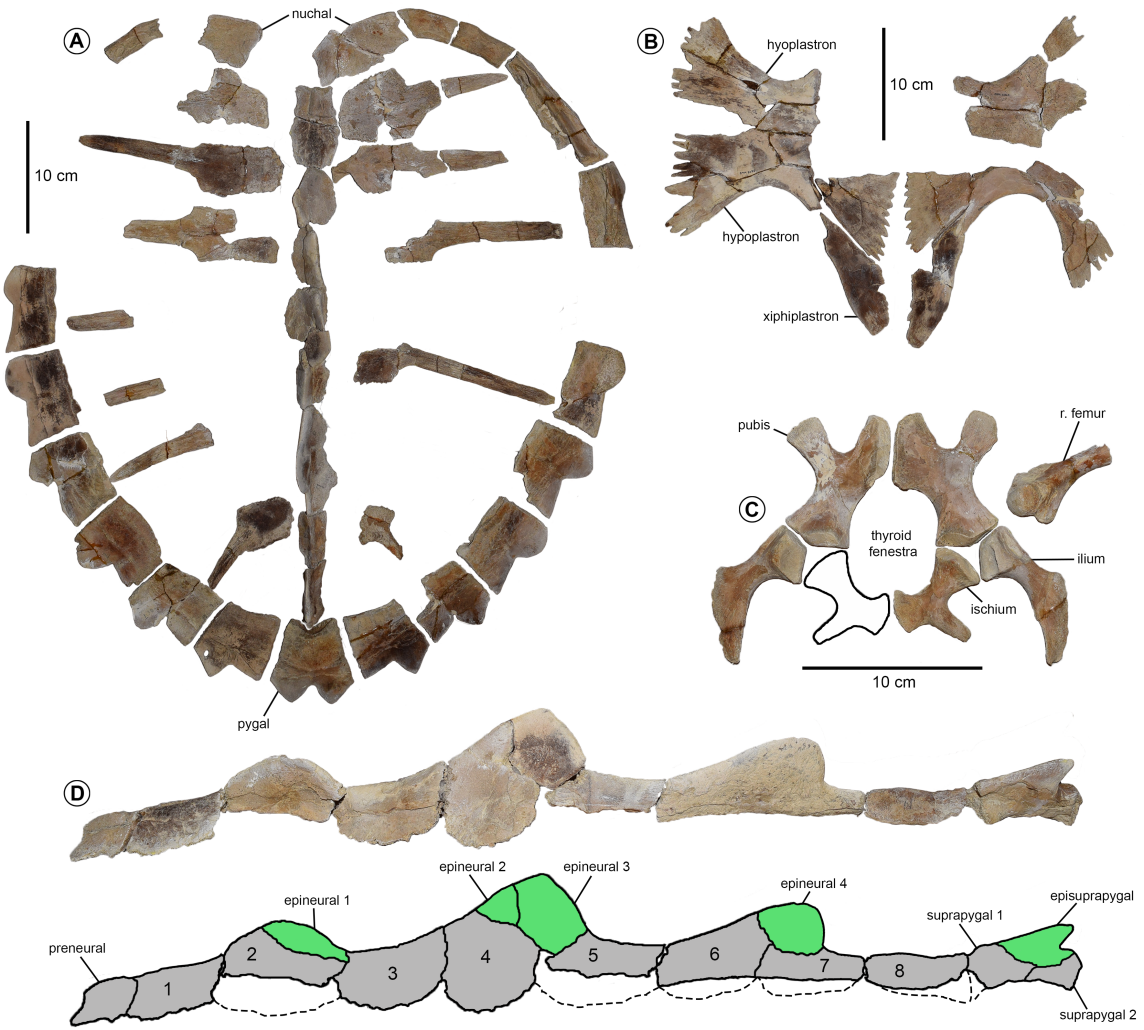
*Prionochelys matutina* (MSC 3036) from the Mooreville Chalk of Alabama. (A) Carapace in dorsal view. (B) Plastron in ventral view. (C) Pelvis in dorsal view. (D) Neural-epineural series in left lateral view.

**Palatal elements** The premaxilla contacts the maxilla laterally and the vomer posteriorly. Ventrally the premaxilla forms the anterior part of the upper triturating surface and along its anterior margin, exhibits a ventrally oriented labial ridge similar to the ridge seen in both *Toxochelys* and *Ctenochelys*. Dorsally the premaxilla contributes to floor of the apertura narium externa (ch. 36/0). Both maxillae are nearly intact and form the anterolateral edge of the skull. The maxilla contacts the premaxilla anteriorly, the vomer anteromedially, and the palatine medially. The posterior contacts of the maxilla cannot be determined. Dorsally the maxilla floors the fossa orbitalis and anteriorly forms the lateral margin of the apertura narium externa. In ventral view, the maxilla serves as the majority of the upper triturating surface and is moderately dorsally concave. The labial ridge of the maxilla is pronounced and diminishes in height posteriorly (ch. 42/0). The palatine is sutured with the vomer and maxilla in ventral view. The lateral edge of the palatine is sutured to the maxilla and forms a relatively small portion of the triturating surface (ch. 39/0). The degree of contribution from the palatine to the triturating surface is slightly greater than that of *Toxochelys latiremis* (Matzke, 2009; USNM 11639, text-fig. 5, p. 105) and is comparable to that of *Ctenochelys acris* (Gentry, 2016; RMM 6157, fig. 4, p. 6). The vomer is only partially preserved but there is enough of the element to determine that it contacted the premaxilla anteriorly (ch. 47/0), the maxilla laterally, and the palatine posterolaterally. Ventrally, it appears as though the vomer contributed significantly to the upper triturating surface (ch. 51/1). The curved posteromedial margin of the preserved section of the vomer is interpreted here as being the anterior margin of the foramen orbito-nasale. Anteroventrally, the lateral most extensions of the vomer are similar to those found in certain specimens of *Toxochelys latiremis* (Matzke, 2009; AMNH 5118, text-fig. 2, p. 101).

**Palatoquadrate elements** Both quadrates are completely preserved with MSC 39030 (Fig. 4). In dorsal view, the quadrate contacts the quadratojugal anterolaterally, the squamosal posteriorly, the opisthotic posteromedially, and the prootic medially. The quadrate fully encloses the anterior perimeter of the antrum postoticum (ch. 55/2) and forms the majority of the processus trochlearis oticum (ch. 57/2, ch. 58/0). Ventrally the quadrate contacts the pterygoid medially, the opisthotic posteromedially, and the squamosal posterolaterally (ch. 59/0). The medial lobe of the condylus madibularis is slightly larger than the lateral lobe, similar to the condition observed in *Ctenochelys acris* (Gentry, 2016; RMM 6157, fig. 4, p. 6) and adult specimens of *Toxochelys latiremis* (Matzke, 2009; AMNH 1042, text-fig. 8, p. 110).

Both pterygoids are nearly intact and in ventral view, contact the quadrate posterolaterally, the exoccipital (ch. 70/1) and basioccipital (ch. 64/1) posteriorly, and the basisphenoid posteromedially. Though badly worn, the sagittal ridge present in some Cretaceous pan-chelonioids along the medial suture of the pterygoids appears to be highly diminished or absent in *Prionochelys matutina* (ch. 74/0). The pterygoid extends posteriorly almost to the level of the mandibular condyle but does not actually contact the medial edge of the condyle (ch. 75/0). The dorsal surface of the pterygoid is largely obscured by the prootic and quadrate but beginning at the level of the anterior most point of the basisphenoid and running posteriorly along the sulcus cavernosus is the laminar epipterygoid (ch. 60/1).

**Braincase elements** The prootic contacts the supraoccipital posteromedially, the opisthotic posteriorly, and the quadrate laterally. The dorsal exposure of the prootic of *Prionochelys matutina* is relatively large (ch. 82/0) and the prootic contributes only slightly to the medial part of the processus trochlearis oticum. In ventral view, the prootic is almost entirely covered by the wing shaped posterolateral expansion of the pterygoid (ch. 52/2). The medial wall of the dorsally oriented foramen stapedio-temporale (ch. 95/0) and the associated medial groove are formed entirely by the prootic (Fig. 4B). The prootic covers much of the dorsal exposure of the basisphenoid however, enough of the basisphenoid is visible to discern several important features including the presence of a thick, rod-like rostrum basisphenoidale (ch. 86/1) and the close association between the two large foramina anterius canalis carotici cerebralis (ch. 92/1). In ventral view, the basisphenoid is an anteriorly oriented triangle that tapers at the posteromedial contact of the pterygoids. A prominent C-shaped crest is present immediately anterior to the concave suture between the basisphenoid and basioccipital (ch. 88/1). The basioccipital extends posteriorly to the occipital condyle in ventral view but forms only the medial 1/3rd of the condyle. The remaining portions of the condyle are formed by posteroventral extensions of the exoccipitals (Fig. 4D). The two foramina nervi hypoglossi are present on the posteriorly oriented facet of the exoccipitals and are exposed in ventral view (ch. 98/0). The exoccipitals floor the foramen magnum in dorsal view but do not meet dorsally.

**Carotid and palatine arteries** The internal carotid artery enters the braincase via the foramen posterius canalis carotici interni (fpcci) and runs along the pterygoid in the canalis carotici interni before splitting into the cerebral and palatine branches (ch. 99/2) similar to the condition observed in other Cretaceous and Paleocene pan-cheloniids (Matzke, 2009; Gentry, 2016; Myers et al., 2018). The fpcci is formed entirely by the pterygoid (ch. 100/1). The cerebral branch exits the braincase through the foramen anterius canalis carotici interni on the dorsal surface of the basisphenoid posterior to the rostrum basisphenoidale and anterior to the dorsum sellae. The ventral surface of the pterygoid is badly worn and the relative size and position of the foramen posterius canalis carotici palatinum cannot be determined.

#### Shell

**Figure 6:**
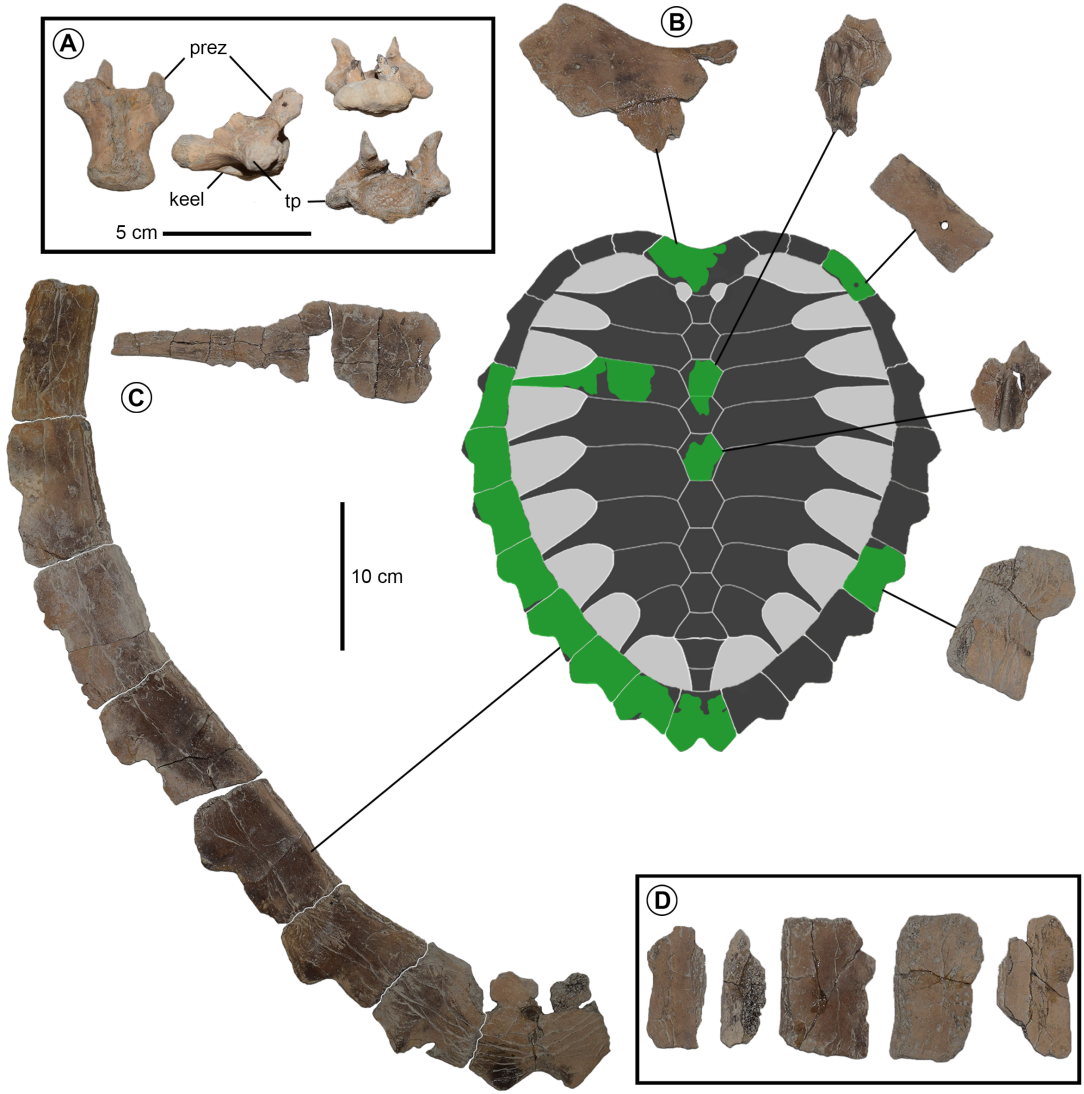
*Prionochelys matutina* (MSC 39013) from the Mooreville Chalk of Alabama. (A) Cervical vertebra in ventral view on the left, right lateral view in the middle, posterior view on the top right, and anterior view on the bottom right. (B) Nuchal in dorsal view. (C) Left peripheral series and 3rd costal in dorsal view. (D) Assorted peripheral fragments in dorsal view.

**Carapace** The carapace of *P. matutina* is strongly cordiform and exhibits the distinctively keeled neural series (Fig. 5; ch. 116/3) and extensively serrated peripherals characteristic of the genus. *P. matutina* is estimated as having a maximum carapace length (MCL) >80 cm based on the estimated total carapace length of the largest known specimen, MSC 39013 (Fig. 6). The anterior margin of the carapace is deeply embayed and the majority of the contribution to this embayment comes from the anterior edge of the nuchal with additional minor contributions from the left and right first peripherals. The posterior margin of the carapace is notched at the pygal with considerable intraspecific variation in the depth and general conformation of this feature.

**Figure 7:**
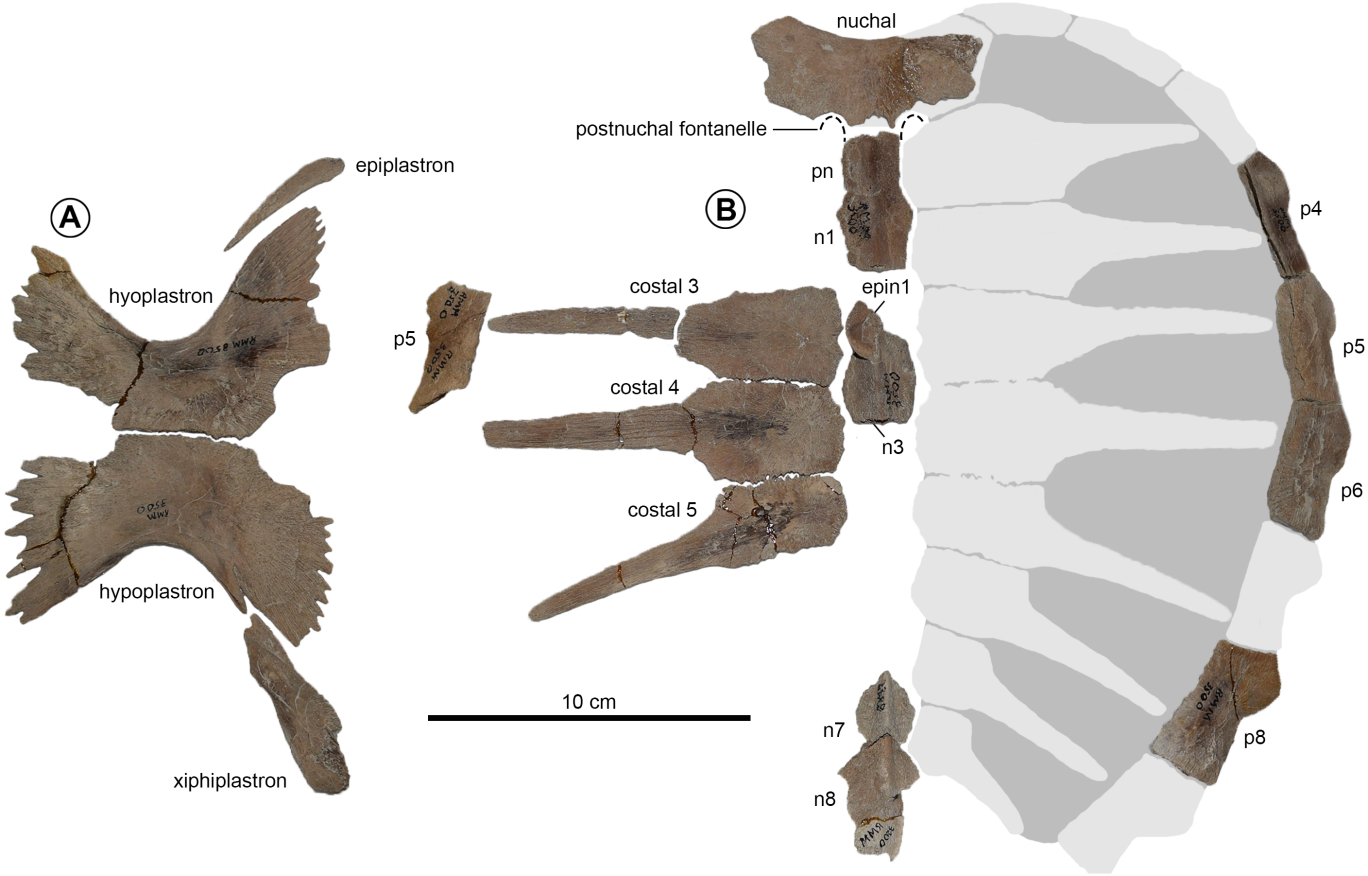
Juvenile *Prionochelys matutina* (MSC3500) from the Mooreville Chalk of Alabama. (A) Preserved plastral elements in ventral view. (B) Carapace in dorsal view. Abbreviations: epin, epineural; n, neural; p, peripheral.

**Peripherals** The highly serrated posterior peripherals characteristic of *Prionochelys* spp. are easily distinguished from those of other closely related Cretaceous pan-cheloniids (i.e., *Ctenochelys* spp. and *Peritresius* spp.) by the sharply pointed ‘blade’ formed by the anterolateral edges of peripherals 6–11 (Figs. 3F, 5A, 6C). There does not appear to be a much ontogenetic variation in the development of the serration as the peripherals of both the smallest (MSC 3500, Fig. 7) and largest (MSC 39013, Fig. 6) individuals are equally serrated. The first peripheral of *P. matutina* is markedly different from that of *P. nauta* with the latter being considerably wider and sector shaped. The second peripheral of *P. nauta* is wider than long, at least in the case of P27454 (Zangerl, 1953; fig. 115, p. 251), as opposed to the second peripheral of *P. matutina* which is much longer than wide. Peripherals 3–11 of *Prionochelys matutina* gradually increase in size and in some of the larger individuals, peripheral 11 can be wider than long (Fig. 6C).

**Figure 8:**
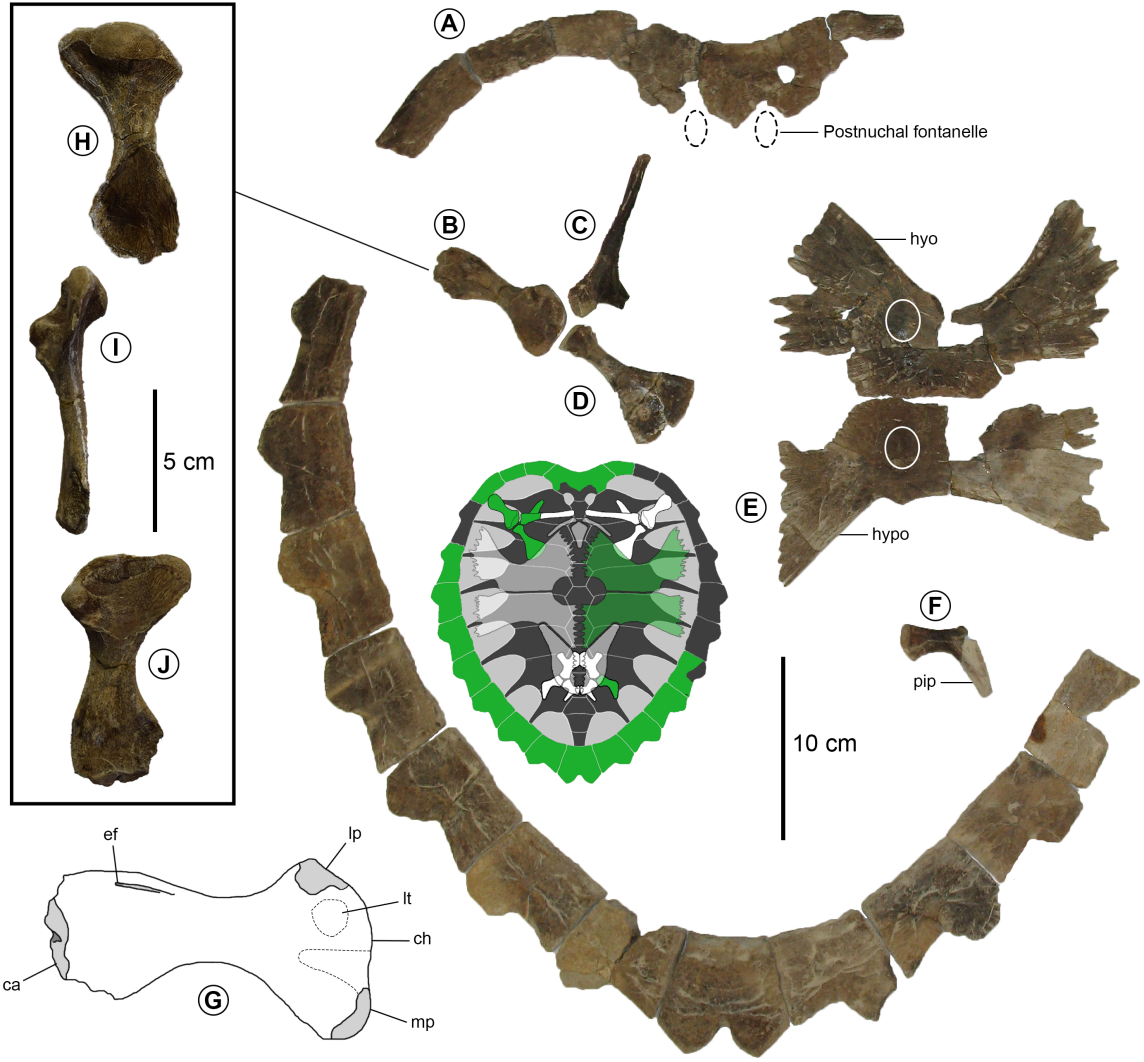
*Prionochelys matutina* (MSC 1540) from the Mooreville Chalk of Alabama. (A) Carapace elements in dorsal view. (B) Humerus in ventral view. (C) Scapula in dorsal view. (D) Coracoid in dorsal view. (E) Plastral elements in ventral view. (F) Ilium in dorsal view. (G) Illustration of humerus in ventral view. (H) Humerus in dorsal view. (I) Humerus in anterior view. (J) Humerus in ventral view. Abbreviations: ca, capitellum; ch, caput humeri; ef, ectepicondylar foramen; hyo, hyoplastron; hypo, hypoplastron; lp, lateral process; lt, m. latissimus dorsi and m. teres major insertion scar; mp, medial process; pip, posterior iliac process. White circles indicate plastral knobs.

**Nuchal** The nuchal of *P. matutina* is nearly three times wider than long (ch. 122/0) and near the posteromedial edge of the ventral surface possesses a raised, elongate pedestal which presumably articulated with the neural spine of the eighth cervical vertebra (ch. 120/2). The posteromedial border of the nuchal shows the same well-developed postnuchal (suprascapular) fontanelles (ch. 123/1; Figs. 6–8) also found in several additional species of Late Cretaceous pan-chelonioid (*Toxochelys latiremis*, *Ctenochelys* spp.). Zangerl (1953) described *Prionochelys* as lacking postnuchal fontanelles and did not include them in his reconstruction of *P. nauta* (Zangerl, 1953, fig. 116, p. 252) though it should be noted that the nuchal of *P. nauta* is unknown. One of the only currently known specimens of *P. nauta*, P26238, does have a nearly intact preneural whose left and right anterolateral margins seem to show evidence of paired embayments possibly indicative of postnuchal fontanelles (Zangerl, 1953, pl. 28). Both juvenile (MSC 3500, Fig. 7) and large adult (MSC 1540, Fig. 8) specimens of *Prionochelys matutina* possess postnuchal fontanelles so this feature is not interpreted as being ontogenetically variable.

**Neurals and epineurals** Preserved with MSC 3036 is the first complete neural series described for any species of *Prionochelys*. The series consists of one preneural, eight neurals, and three epithecal ossifications (epineurals) located at the junctions of neurals 2–3, 4–5, and 6–7 (ch. 126/1, ch. 127/1; Fig. 5D). Zangerl described *Prionochelys* as having nine neurals, not including the preneural, but none of the currently recognized specimens of *Prionochelys* spp. have more than eight. The neurals and epineurals of *P. matutina* exhibit the same undulating midsagittal keel seen in both Marlbook Marl and Niobrara *Prionochelys* material (ch. 127/1). The keeled preneural of *P. matutina* lacks the epineural and anterolateral ‘wings’ thought to be present on the preneural of *P. nauta* (Zangerl, 1953, fig. 114, p. 250) and more closely resembles the preneural of *Ctenochelys acris* Zangerl, 1953 (Zangerl, 1953, fig. 112, p. 244). Based on the arrangement of the neural/epineural series of MSC 3036, it is possible that due to very incomplete nature of previously referred specimens of *Prionochelys nauta* and the inherent difficulty in accurately reconstructing an organism based on such partial remains, the broadly flared preneural and cervical elevation originally figured for *Prionochelys nauta* (Zangerl, 1953; FMNH P26237, fig. 114, p. 250) are, in fact, the nearly triangular 8th neural and the anterior-most portion of the anal elevation found in both *Prionochelys matutina* (Fig. 5D) and *Ctenochelys stenoporus* (Matzke, 2007; USNM 357166, text-fig. 12, p 683)*.* More complete specimens of *P. nauta* are needed to confirm this supposition.

The pectoral elevation of *Prionochelys matutina* is formed by neurals 3–5 and by two epineurals (Fig. 5D). The presence of this second epineural at the pinnacle of the pectoral elevation is a potential autopomorphy of *Prionochelys matutina* and is not exhibited by any other species of pan-cheloniid that possesses epineurals.

**Costals** The costal plates of *P. matutina* are highly reduced resulting in the exposure of the distal rib ends of every costal (ch. 133/3). The costal fontanelles of *P. matutina* are more than 50% of the width of the adjacent costals even in the larger adult specimens (Figs. 5A and 6C) so it is presumable that at no point during this species’ ontogeny does it show the degree of ossification found in larger individuals of *P. nauta* (Zangerl, 1953, fig. 116, p. 252 & pl. 27). The rib end of the first costal inserts into peripheral three, the second costal into peripheral four, and so on until the 10th peripheral which receives the distal rib end of the eighth costal. There is no insertion point visible on the medial facet of the 11th peripheral creating rib-free peripherals anterior and posterior to the costals (ch. 134/0).

**Pygal and suprapygals** Zangerl (1953) originally described *Prionochelys* spp. as having either a single suprapygal or none at all and hypothesized that in *Prionochelys matutina*, the suprapygal had essentially assumed the position and shape of the ninth neural found in other Cretaceous marine turtles. MSC 3036 clearly possesses two suprapygals (ch. 138/1) with the first suprapygal being the larger of the two (ch. 139/1, Fig. 5). The laterally expanded anterior half of the first suprapygal is sutured to the posterior margin of the triangular 8th neural similar to the arrangement of these elements in the type specimen of *Prionochelys galeotergum* (Zangerl, 1953; PR 125, fig. 121, p. 259). Covering much of the dorsal surface of both suprapygals of MSC 3036 is an episuprapygal that extends posteriorly nearly to the junction of the second suprapygal and pygal (Fig. 5D). The apex of the episuprapygal is more pointed and dorsally elevated than that of *Ctenochelys stenoporus* (Matzke, 2007; text-fig. 13B, p. 685) and *Peritresius ornatus* (Baird, 1964; NJSM 11051, fig. 5, p. 16). There is considerable variation among referred *Prionochelys* specimens in the degree to which the posterior margin of the pygal is excavated however, *Prionochelys* pygals are all deeply notched posteriorly (ch. 141/0).

**Plastron** The plastron associated with MSC 3500 is the most complete *Prionochelys matutina* plastron yet described and the first juvenile plastron identified for any species of *Prionochelys* (Fig. 7A). The length of the plastron in nearly double the width and the general arrangement of the plastral elements closely conforms to the pattern observed in other Cretaceous pan-cheloniids in having proportionally diminutive, medially sutured epiplastra anterior to the entoplastron (ch. 161/1), well-developed lateral and central plastral fontanelles (ch. 150/1) created by a reduction in the width of the hyo-hypoplastral suture (ch. 154/1), and narrow, elongate xiphiplastra (ch. 170/2) with S-shaped lateral margins. The axillary process of the hyoplastron appears to have slightly contacted the carapace (ch. 163/0) near the level of the 4th peripheral (ch. 164/2) and does not extend anteriorly beyond the margin of the anteromedial process of the plastron. The inguinal process of the hypoplastron also seems to have slightly contacted the peripherals (ch. 167/0) near or at the level of the 7th peripheral (ch. 168/1). Distinct knobs are present on the ventral surface of the hyo-, hypo-, and xiphiplastra in virtually identical locations to those found in *Ctenochelys stenoporus* (Matzke, 2007; USNM 357166, text-fig. 13, p. 685).

#### Appendicular skeleton

**Pectoral girdle** The right half of the shoulder girdle is preserved with MSC 1540 and represents the first scapula, coracoid, and humerus described for this genus (Figs. 8B–8D). The angle formed by the acromial and scapular prongs of the scapula is approximately 110–115° (ch. 220/1) making it nearly equivalent to the angle found in *Ctenochelys stenoporus* (Matzke, 2007; USNM 357166, text-fig. 14, p. 686) and *Ctenochelys acris* (Gentry, 2016; MSC 35085, fig. 8, p. 13). Only the base of the scapular process is present but the acromial process is intact and has a length of 6.8 cm. There is a clear glenoid neck (ch. 218/1) but it is somewhat reduced compared to the same feature of *Toxochelys* spp. or *Ctenochelys acris*. The coracoid is the same length as the humerus (ch. 221/1) and is interpreted here as being 7.6 cm long with a posteromedial plate that measures 4.6 cm across. The coracoid of *Prionochelys matutina* is relatively shorter than any other species of Cretaceous pan-cheloniid. The posteromedial plate of the coracoid of *P. matutina* is proportionally larger than *Ctenochelys stenoporus* or *Toxochelys latiremis* with the minimal width of the coracoid diaphysis (1.0 cm) contained 4.6 times in the maximum width of the posteromedial plate. In *Toxochelys latiremis* (Zangerl, 1953), the comparable value is 3.6 and is almost ∼4.0 for *Ctenochelys stenoporus* (Matzke, 2007). The proportions of the coracoid of *P. matutina* are much closer to those of *Toxochelys moorevillensis* (Zangerl, 1953; FMNH PR136, p. 191) with its comparable value of 4.8 and *Ctenochelys acris* (Gentry, 2016; fig. 8, p. 13) where the minimal width of the coracoid diaphysis is contained nearly 5 times in the width of the posteromedial plate.

**Humerus** The humerus of MSC 1540 is completely preserved and is 7.5 cm in length (Fig. 8B, 8G–8J). The proximal end of the humerus is 4.7 cm wide (ch. 244/0). The medial process extends laterally further from the sagittal diaphyseal midline than the same feature of *Ctenochelys stenoporus* (Matzke, 2007; USNM 357166, text-fig. 14, p. 686), the most closely related pan-cheloniid for which the humerus is known. The medial process is much larger than the lateral one. The rounded lateral process of the humerus of *P. matutina* (ch. 240/0) is located only slightly distal to the proximal articular surface (ch. 238/1).

**Pelvis** The pelvic elements of *Prionochelys matutina* are largely indistinguishable from the same elements of *Ctenochelys stenoporus* (Matzke, 2007; USNM 357166, text-fig. 15, p. 686) with only a few notable exceptions. The thyroid fenestra is coalescent (ch. 225/0) and shows no indication of even a partial subdivision by the medial processes of the pubes or ischia (Fig. 5C). The elongate iliac neck (ch. 226/1) is slightly shorter than that of *Ctenochelys stenoporus* but without a larger sample size of pelvic elements belonging to both species, it is difficult to determine how much of this difference may be due to interspecific or intraspecific variation. The prominent lateral process of the pubis is flat (ch. 231/1) and extends anteriorly to the level of the anterior margin of the medial pubic process. This is in contrast to *Ctenochelys stenoporus* where the medial pubic process extends anteriorly beyond the level of the lateral process. The metischial process of the ischium is present (ch. 234/1) and appears to have reached further posteriorly than the metischial process of *Ctenochelys stenoporus.*

**Vertebrae** A single cervical vertebra is preserved with MSC 39013 and represents the first vertebrae of any kind described for *Prionochelys* (Fig. 6A)*.* The transverse processes are located near the anterior end of the centrum (ch. 187/1) and a distinct ventral keel is present (ch. 188/1) spanning nearly the full length of the centrum. The prezygophyses extend anteriorly beyond the level of the anterior margin of the centrum. Both postzygophyses are missing. The vertebra is clearly procoelous (ch. 191/1) and though the vertebra is badly worn, there appear to have been two distinct articular surfaces on the anterior central articulation and although it is unclear exactly which cervical vertebra this would have been (1–8), this feature does indicate the presence of a double articulation at some point in the cervical series.

**Figure 9:**
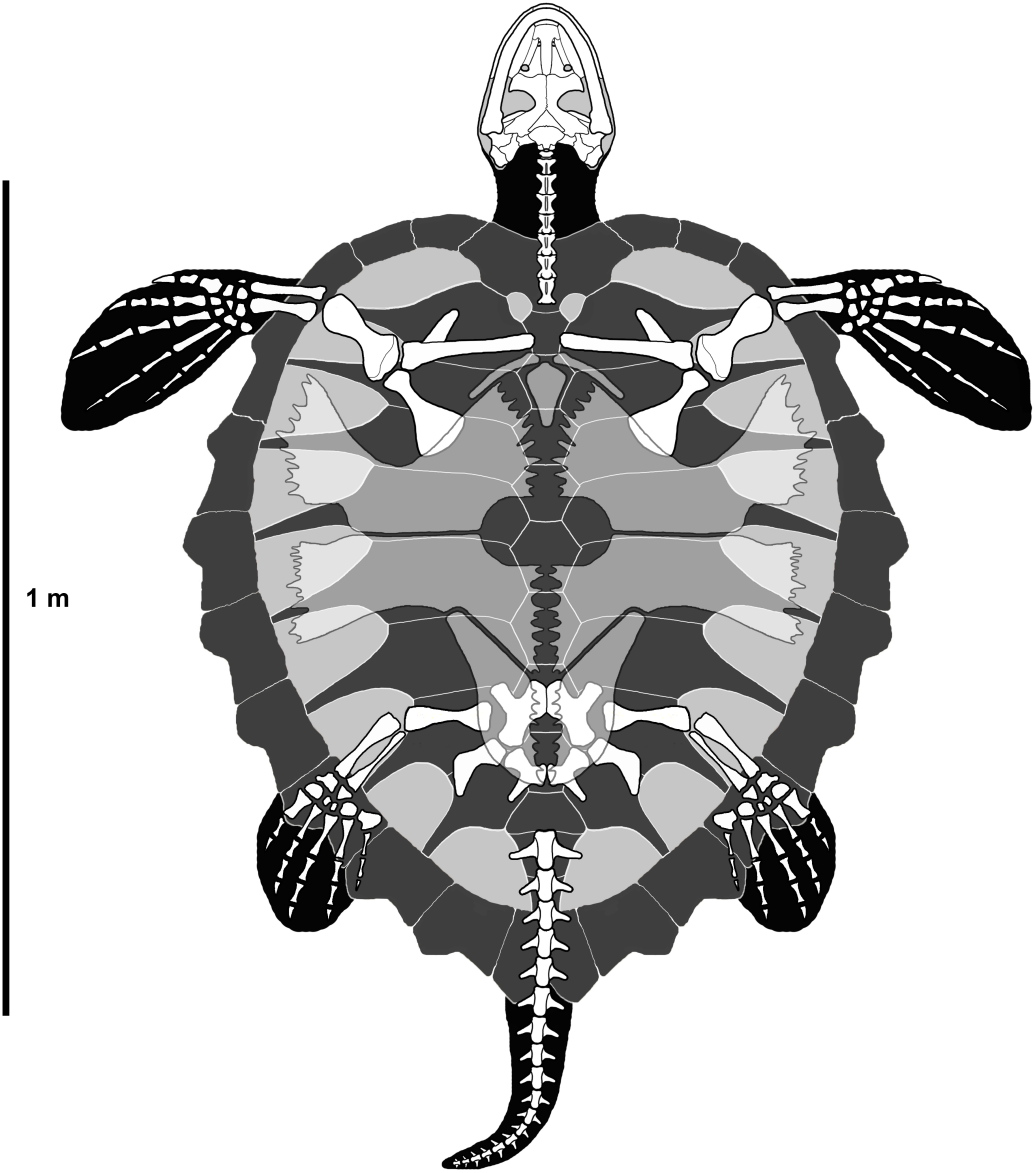
Reconstruction of *P. matutina* in ventral view.

### Ontogeny of *Prionochelys*

**Figure 10:**
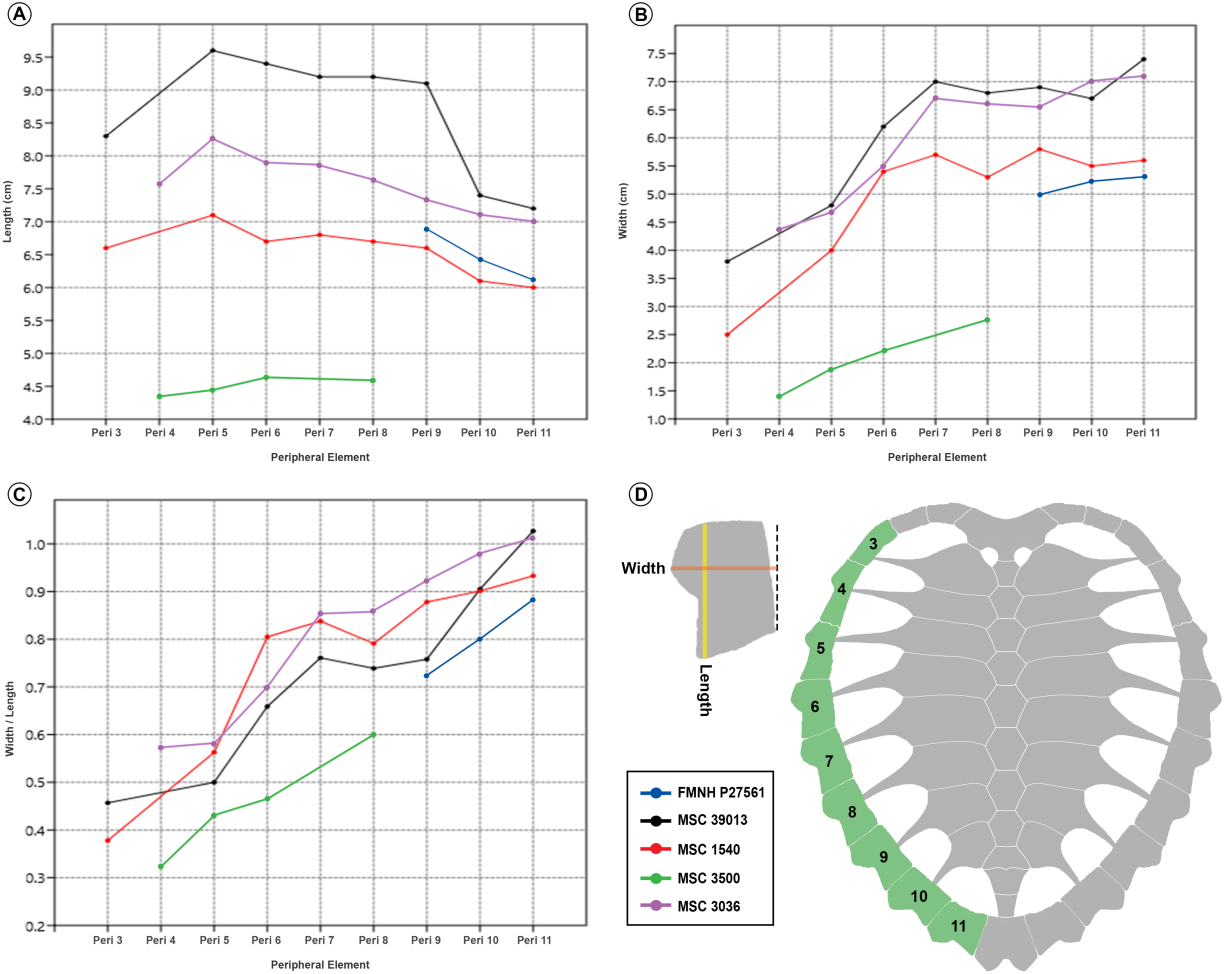
*Prionochelys matutina* peripherals 3–11 growth metrics. (A) Length measurements. (B) Width measurements. (C) Width as a function of length (width/length). (D) Diagram showing the method for metric data collection from each peripheral element on the left and the specific elements used for comparison on the right.

**Figure 11:**
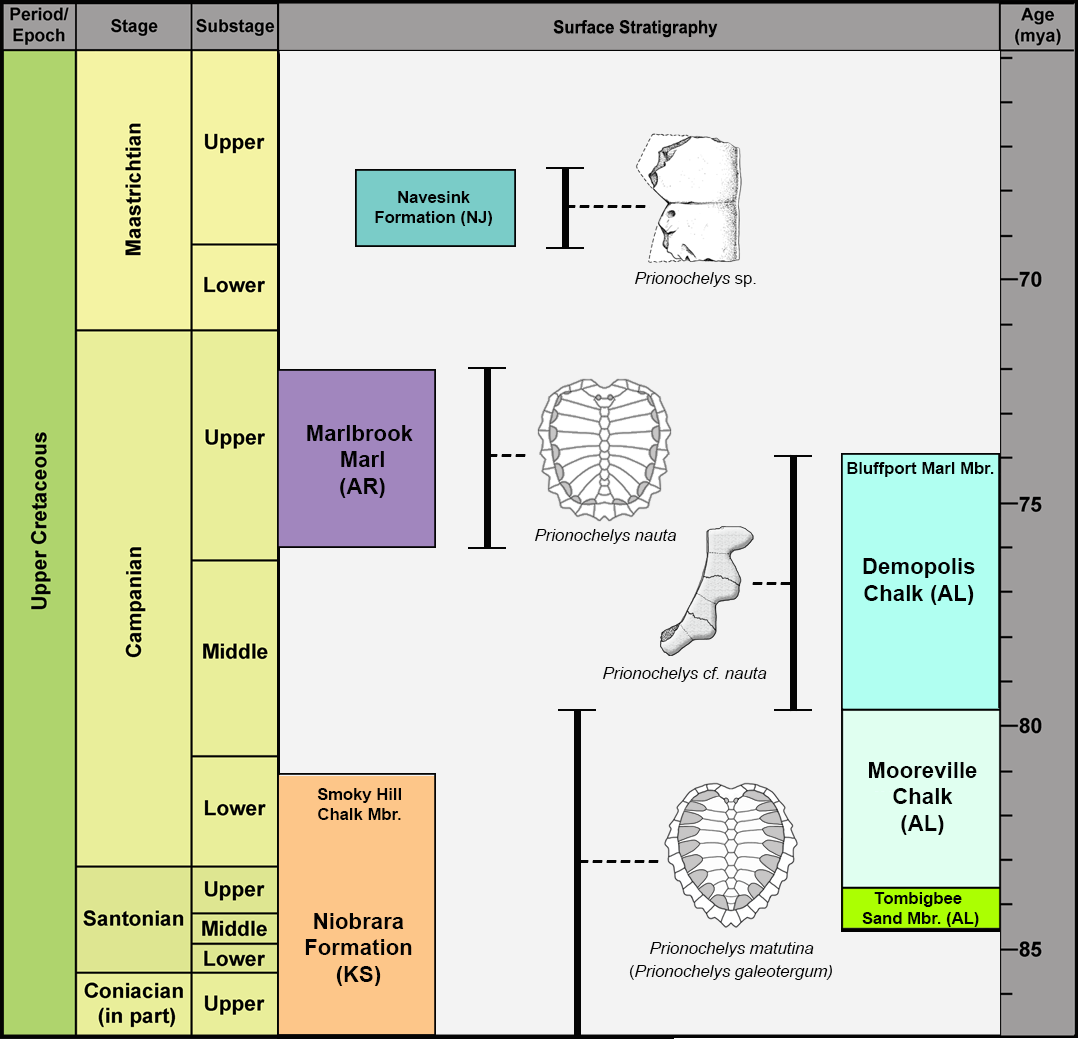
Stratigraphic occurrence of *Prionochelys*. Illustration of *Prionochelys* sp. peripheral from Baird & Case (1966).

**Figure 12:**
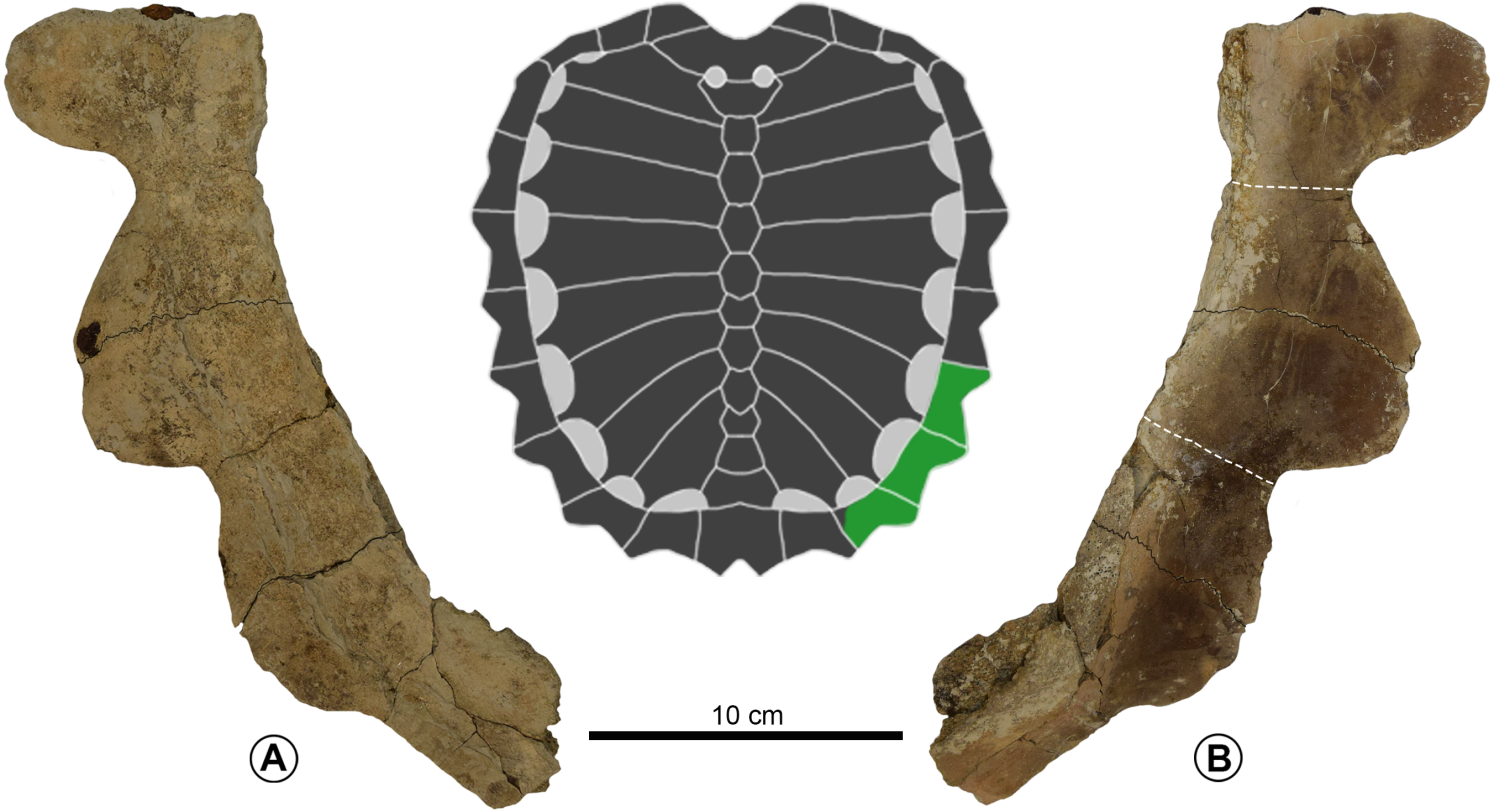
*Prionochelys cf. nauta* (ALMNH 6673) from the Demopolis Chalk of Alabama. Peripherals 8–10 in ventral (A) and dorsal (B) views. Dashed lines indicate scute sulci.

**Figure 13:**
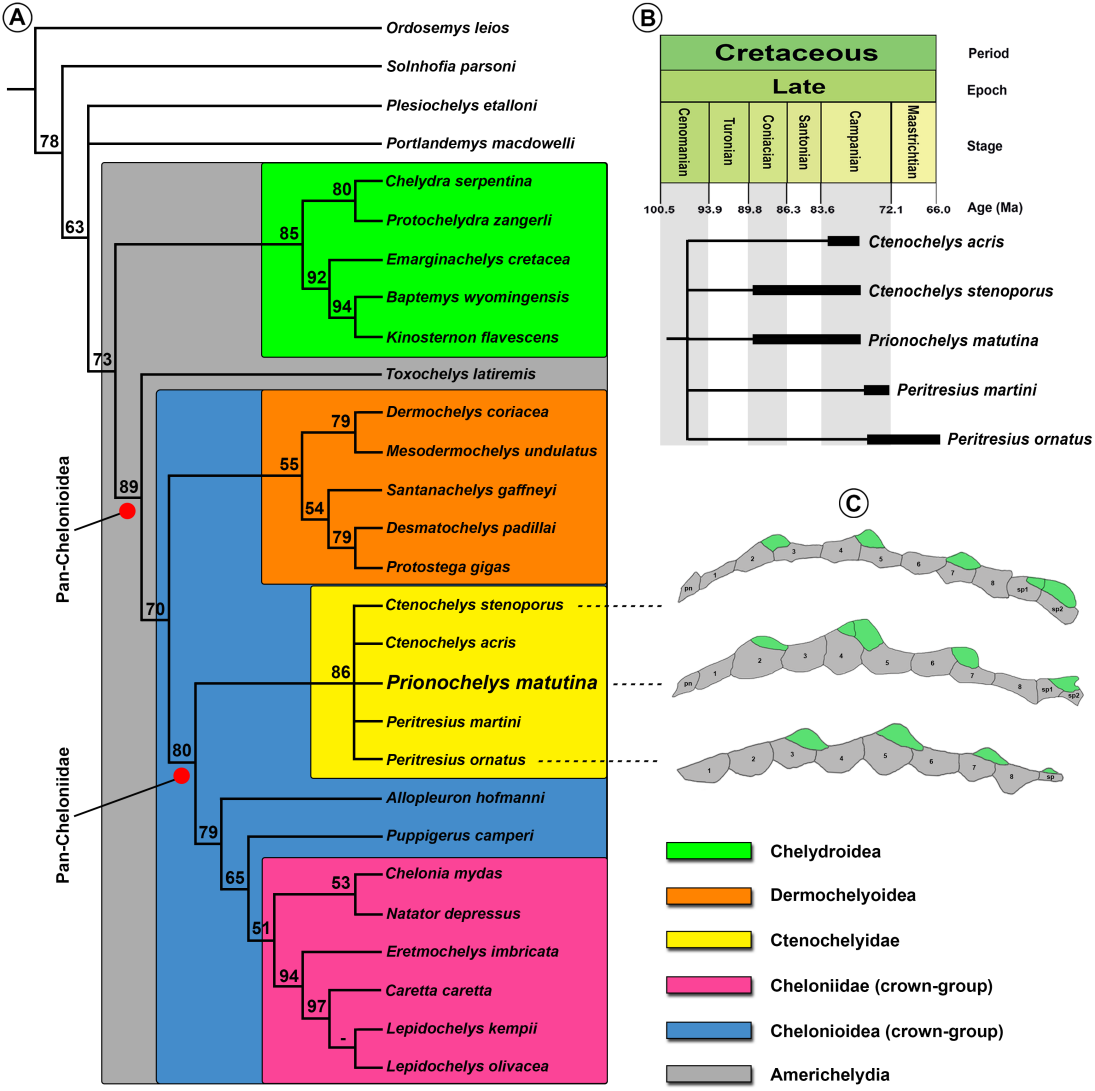
Phylogenetic hypothesis. (A) Strict consensus of 42 MPT’s (CI: .645; RI: .719) showing the position of *Prionochelys matutina* within Americhelydia. Numbers indicate bootstrap values. Bootstrap values of 100 are represented by dashes. (B) Cladogram of Ctenochelyidae showing the fossil occurrence of each included species. (C) Comparison of the general arrangement of the neural-suprapygal series of *C. stenoporus*, *P. matutina*, and *Peritresius ornatus* with epithecal elements highlighted in green. Abbreviations: pn, preneural; sp, suprapygal.

**Figure 14:**
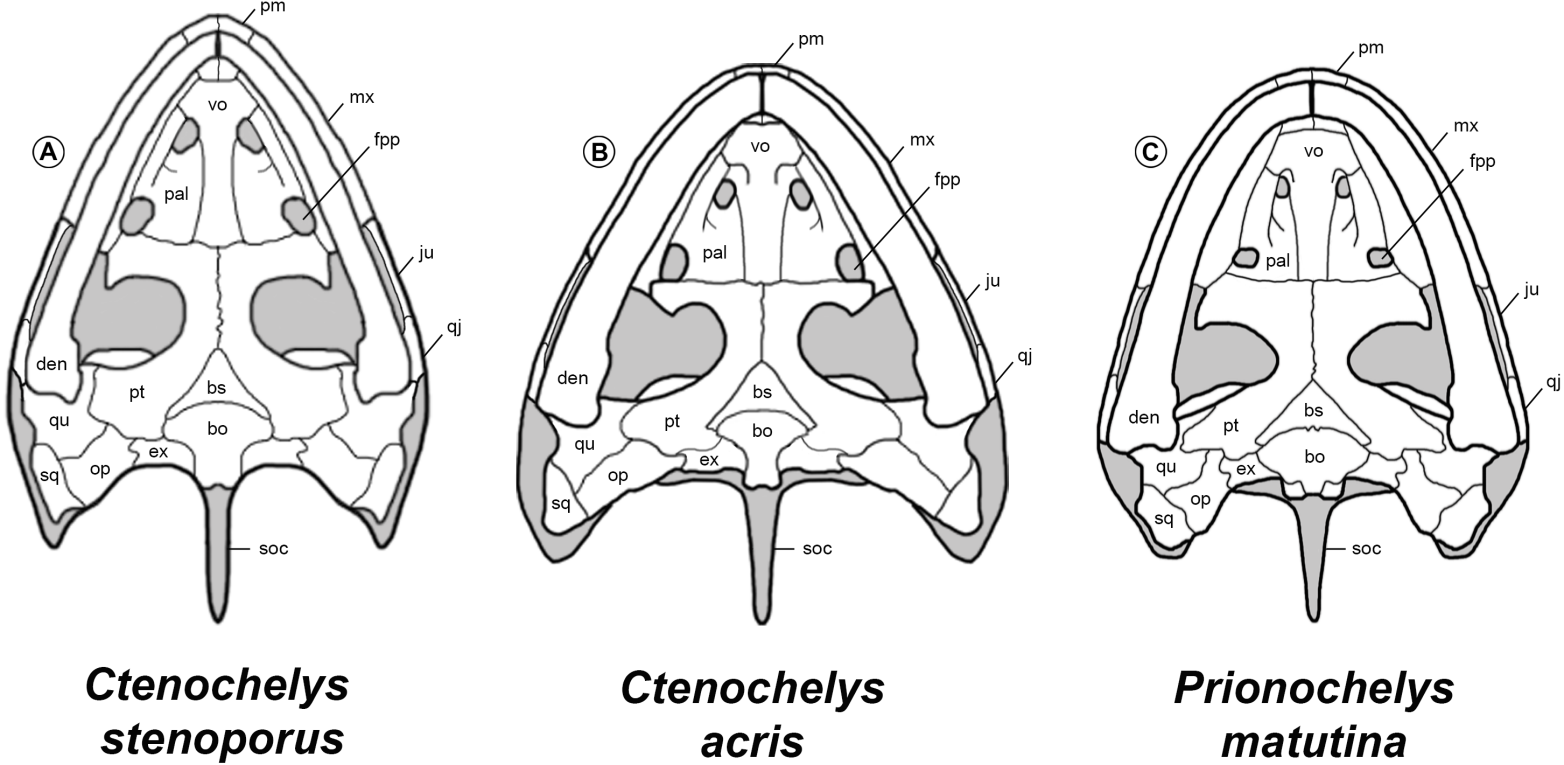
Comparison of the skulls of *Ctenochelys* and *Prionochelys* in ventral view. Abbreviations: bo, basioccipital; bs, basisphenoid; den, dentary; ex, exoccipital; fpp, foramen palatinum posterius; ju, jugal; mx, maxilla; op, opisthotic; pal, palatine; pm, premaxilla; pt, pterygoid; qj, quadratojugal; qu, quadrate; soc, supraoccipital crest; sq, squamosal; vo, vomer.

**Figure 15:**
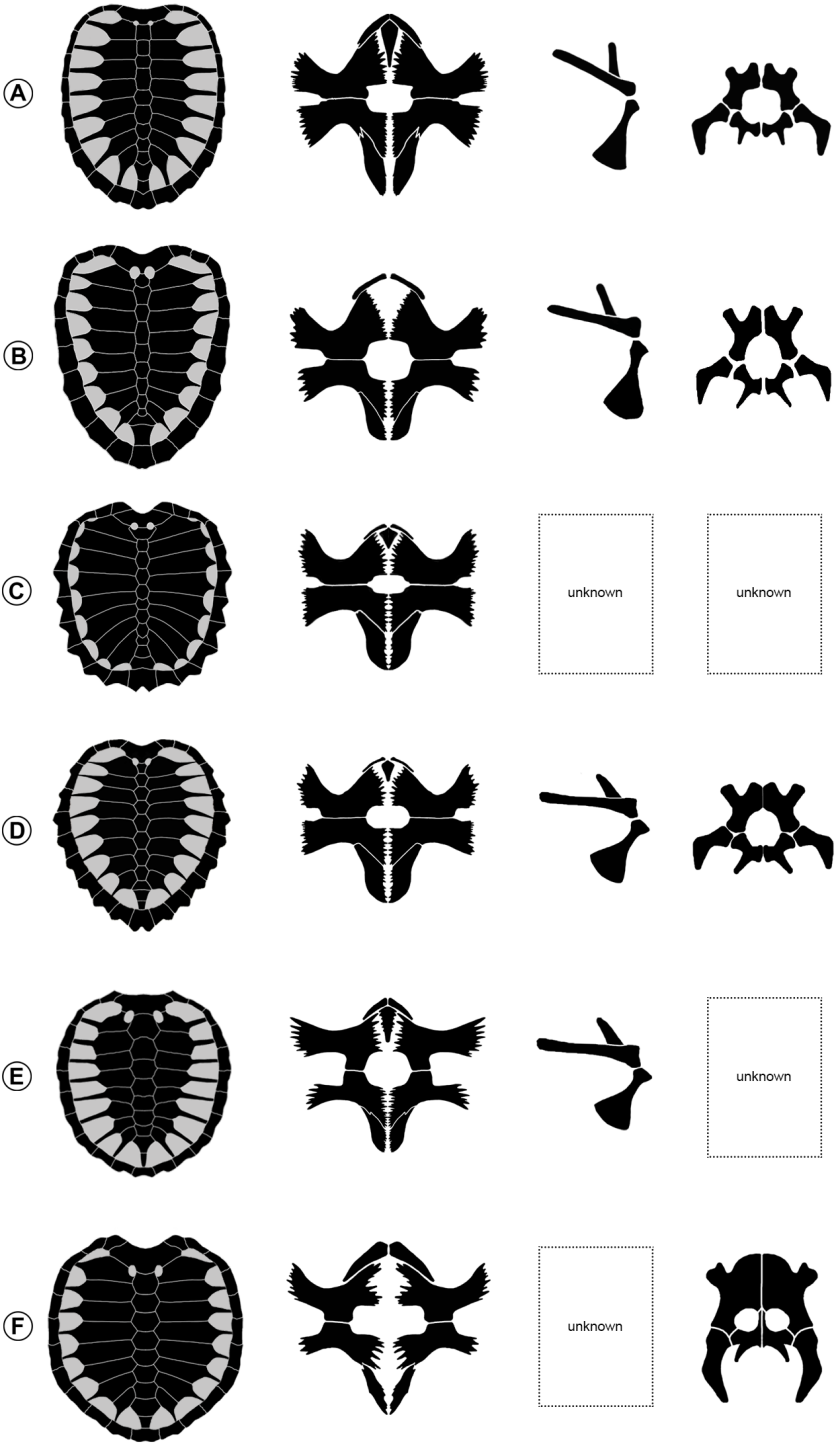
Carapace, plastron, shoulder girdle, and pelvis of ctenochelyid turtles in ventral view. (A) *Ctenochelys stenoporus* (=*Toxochelys bauri*), YPM 1786, after Wieland, 1905. (B) *Ctenochelys acris*, MSC 35085, after Gentry, 2016 with modification to show correct orientation of scapula. (C) *Prionochelys nauta*, FMNH P26237 & FMNH P26238, after Zangerl, 1953. (D) *P. matutina*, MSC 3036 & MSC 1540. (E) *Peritresius ornatus*, NJSM 11051, after Baird, 1964. (F) *Peritresius martini*, ALMNH 6191, after Gentry et al., 2018. The entoplastron of *Ctenochelys acris* and *Peritresius martini* are unknown. Not to scale.

Ontogenetic variation is virtually unknown for species of fossil chelonioid due primarily to a lack of described juvenile specimens for many species. MSC 3500 represents the first formally described juvenile of *P. matutina* and one of only three described juvenile Cretaceous pan-chelonioid specimens (Zangerl, 1953; KUVP 1244, pl. 21, p. 164; Matzke, 2007; USNM 357166, text-figs. 1–15). The general conformation of the carapace of MSC 3500 (Fig. 7B) compares closely to that of adult specimens of *P. matutina* (Fig. 9). The adult nuchal emargination is considerably more deeply embayed and the width of the contact between the nuchal and first peripheral is much greater in adult specimens. Peripherals 4–8 are always longer than wide in both adults and juveniles, however, it appears that in juvenile and sub-adult individuals, these elements primarily increased in length (Fig. 10A) until the individual reached a certain age at which point the peripherals increased more in width than in length (Figs. 10B and 10C). This increase in the width of the peripherals seems to continue throughout the life of the animal and in the largest specimens reported here, the width of peripheral l1 actually exceeds its length (Fig. 10C).

The plastral fontanelles of *P. matutina* appear to decrease in size during growth. The smallest currently known juvenile (MSC 3500) possesses a central plastral fontanelle whose length is equal to that of the hyo-hypoplastral contact. In the largest adult specimen where this feature is preserved (MSC 1540), the length of the central plastral fontanelle is only ∼65% of the width of this contact. In contrast with the plastral fontanelles, the postnuchal fontanelles either stay proportionally equivalent or slightly increase in size during ontogeny. Unlike Matzke (2007) observations of *Ctenochelys stenoporus*, the lateral carapacial fontanelles of *P. matutina* do not decrease in size during growth given that the proportions of these features in both adults and juveniles seem to be nearly equivalent. There do not appear to be any other significant ontogenetic changes in the proportions of the plastron or carapace, however more complete juvenile specimens may reveal additional ontogenetic variation.

### Taxonomy of *Prionochelys*

Three original species of *Prionochelys* were recognized based on geographic location, stratigraphic age, and minor variations in the arrangement of the neural/epineural series (Zangerl, 1953). *P. galeotergum* is based on a single specimen from the Niobrara formation of Kansas (FMNH PR125) and was differentiated from *P. matutina* and *P. nauta* on the lack of contribution to the pectoral keel elevation by the 2nd or 3rd neural, the presence of a keel elevation dorsal to the suprapygals, and the blunt, narrow marginal facet of the pygal. Examination of the type of *P. galeotergum* shows that the 2nd neural does contribute slightly to the pectoral keel elevation similar to the condition observed in *P. matutina*. Zangerl (1953) identified the epithecal element preserved with PR125 as the epineural between the 5th and 6th neurals, despite the fact that neither the 5th nor 6th neural are preserved with this specimen. The presence of an episuprapygal ossification was the second character used by Zangerl to diagnose *P. galeotergum* from other species of *Prionochelys* but at the time of his assessment, PR125 was the only *Prionochelys* specimen for which this area of the carapace was adequately known. The newly described specimens of *Prionochelys* presented here show that this feature was also present in *P. matutina*. The lone epithecal element with PR125 is identical to the episuprapygal of *P. matutina* and when turned backwards from the direction figured by Zangerl, articulates nicely with the small sutural pit on the anterior facet of the pygal.

The third feature, the size and shape of the pygal, is clearly a variable character for all species of *Prionochelys*. The anterior facet of the pygal expands laterally during ontogeny as evidenced by the differences between the pygal of the smaller specimens of *P. matutina* (MSC 3036, Fig. 5; MSC 1540, Fig. 8) and that of the larger specimen MSC 39013 (Fig. 6). Zangerl (1953) notes that due to its small size, PR125 likely represents a younger individual so it is presumable that the anterior facet of the pygal will be narrow when compared with larger specimens of *Prionochelys*. Based on the characters outlined above, *P. galeotergum* should be considered a junior synonym of *P. matutina.*

The other species of *Prionochelys*, *P. nauta*, is notably distinct from *Prionochelys matutina* while still exhibiting all of the apomorphic characters of the genus. Zangerl described the morphology of *P. matutina* as being more ‘primitive’ than that of *P. nauta* and proposed a direct ancestor/descendant relationship between the two species. The fossil occurrence of the two species supports this notion with *P. matutina* being the stratigraphically older member of the lineage (Fig. 11). Unfortunately, the anatomical data available for *P. nauta* is extremely limited and without more complete specimens of *P. nauta*, this proposed evolutionary relationship cannot be tested within a phylogenetic context. Despite the lack of described material for this species, *P. nauta* possessed the same midsagittal carapacial keel comprised of both neurals and epineurals found in species of both *Ctenochelys* and *Peritresius*. There are two clearly diagnosable species of *Prionochelys*, and the following revision of the genus is proposed:

**Table:** 

TESTUDINES Batsch, 1788
CRYPTODIRA Cope, 1868
AMERICHELYDIA Joyce et al., 2013
CHELONIOIDEA Oppel, 1811
PAN-CHELONIIDAE Joyce, Parham & Gauthier, 2004
*PRIONOCHELYS*Zangerl, 1953

**Type species** *Prionochelys nauta* Zangerl, 1953

**Amended generic diagnosis** Moderately sized marine turtle similar in size to *Ctenochelys stenoporus.* Differentiated from *Toxochelys* Cope, 1873 and *Thinochelys* Zangerl, 1953 by the presence of keeled neurals, epineurals, and strongly serrated posterior peripherals. Diagnosed from other Cretaceous pan-cheloniids such as *Ctenochelys* spp. by the highly serrated lateral edges of peripherals 4–11 and the deeply embayed anterior nuchal emargination resulting in a more distinctly cordiform carapacial outline. Diagnosed from *Peritresius* spp. by the increased contact between the hyo- and hypoplastron, the relative width of the neuralia, and the presence of eight neurals and one preneural.

**Table:** 

*PRIONOCHELYS MATUTINA*Zangerl, 1953
Figs. 3–9

**Synonymy** *Prionochelys galeotergum* Zangerl, 1953; *Prionochelys matuina* [sic] Hirayama, 1997

**Holotype** FMNH P27561, Fig. 3; Zangerl, 1953, fig. 118, p. 255.

**Type locality** Mooreville Chalk Formation, Dallas County, Alabama

**Referred material** FMNH specimens: P27561, PR31, P27479, PR222, PR185; MSC specimens: MSC 3500, MSC 6086, MSC 3036, MSC 2720, MSC 1915, MSC 39013, MSC 38604, MSC 2610, MSC 2045, MSC 3140, MSC 1719, MSC 5626.

**Amended diagnosis** MCL ∼90–100 cm. Specimens can be diagnosed as *Prionochelys matutina* based on the presence of a pair of epineurals between neurals 4–5, the loss of a cervical keel elevation, and the sharply crested, posteriorly oriented apex of the episuprapygal.

**Table:** 

*PRIONOCHELYS NAUTA*Zangerl, 1953
Fig. 12

**Synonymy** None.

**Holotype** FMNH P26237, (Zangerl, 1953), pl. 27.

**Type locality** Marlbrook Marl Formation, Howard County, Arkansas, USA.

**Referred material** ALMNH 6673, Fig. 12; for Marlbrook Marl specimens, see Zangerl, 1953, p. 249.

**Diagnosis** Can be distinguished from *P. matutina* by the presence of a nearly triangular first peripheral in dorsal or ventral view, a pronounced cervical keel elevation on the dorsal facet of the preneural, a preneural that is wider than long with distinct anterolateral ‘wings’, the lack of a second epineural integrated into to the pectoral elevation of the neural series, peripherals with a deeply notched marginal edge such that the depth of the notch in peripherals 6–10 is equal to or greater than the width of the peripheral at the level of the scute sulcus, and costal plates wider than the adjacent fontanelles.

**Description** See Zangerl (1953).

## Phylogenetic Analysis

Parsimony analysis resulted in 42 MPTs of 342 steps. A strict consensus tree (Fig. 13A) shows good resolution of the various major clades within Americhelydia. *Toxochelys latiremis* was recovered as the most primitive member of Pan-Chelonioidea while *Prionochelys matutina* was recovered as a pan-cheloniid and member of a monophyletic clade which also includes *Ctenochelys* and *Peritresius* (Fig. 13B). This grouping is sister to the clade originating from the common ancestor of *Allopleuron hofmanni* (Gray, 1831), *Puppigerus camperi* (Gray, 1831), and Cheloniidae. The possibility of *Prionochelys*, *Ctenochelys*, and *Peritresius* forming a monophyletic clade was first proposed by Hirayama (1997) based on the presence of epithecal ossifications (epineurals) dorsal to the neurals (ch. 127/1). The number of epineurals, their position within the neural series, and the general morphology of the individual epineurals varies greatly within the group (Fig. 13C). The loss of a cervical keel elevation and the sharply pointed, posteriorly hooked apex of episuprapygal are both apomorphies of *Prionochelys* and the presence of a second epineural in the formation of the pectoral elevation is interpreted here as an autapomorphy of *P. matutina*. The synapomorphies uniting the major clades within Pan-Chelonioidea are summarized below.

**Table:** 

PAN-CHELONIOIDEA Joyce, Parham & Gauthier, 2004

Originating with the common ancestor of *Toxochelys latiremis* and *Lepidochelys kempii* (Garman, 1880), this clade is united by an open suture between the squamosal and quadrate (ch, 30/1), an articulation between the nuchal and the neural spine of the 8th cervical vertebra in the form of a raised pedestal on the ventral midline of the nuchal (ch. 120/2), the retention of a posterior plastral fontanelle in the adult stage (ch. 151/1), xiphiplastra being narrow, posteriorly elongate struts (ch. 170/2), humeri at least as long as the femora in length (ch. 246/1), and a flattening of the carpal and tarsal elements (ch. 254/1).

**Table:** 

CHELONIOIDEA Oppel, 1811

Originating with the most recent common ancestor of *Dermochelys coriacea* (Vandellius, 1761) and *Chelonia mydas* (Linnaeus, 1758), crown group chelonioids are united here by a concealed foramen stapedio-temporale in dorsal view (ch. 17/0), the presence of a rod-like rostrum basisphenoidale (ch. 86/1), and having a coracoid that meets or exceeds the length of the humerus (ch. 221/1).

**Table:** 

PAN-CHELONIIDAE Joyce, Parham & Gauthier, 2004

Recovered in the present study as a paraphyletic assemblage of Cretaceous and Tertiary species, Pan-Cheloniidae is supported by the presence of an incipient secondary palate (ch. 40/1), a deep C-shaped concavity on the ventral surface of the basioccipital (ch. 81/1), a high dorsum sellae (ch. 90/1), having foramina anterius canalis carotici cerebralis smaller than the foramina anterius canalis carotici palatinum (ch. 91/1), and the entry point of the palatine artery into the skull being the foramen posterius canalis carotici palatinum between the basisphenoid and pterygoid (ch. 101/1).

**Table:** 

CTENOCHELYIDAE (newly proposed clade)

The existence of this taxon has been hypothesized for more than 20 years (Hirayama, 1997) and is supported by phylogenetic evidence in the present study and in other cladistic studies of marine turtles incorporating multiple species of Late Cretaceous pan-cheloniid (Gentry et al., 2018). I propose the formal naming of this group as Ctenochelyidae after the stratigraphically oldest member of the group, *C. stenoporus.*

**Table:** 

TESTUDINES Batsch, 1788
CRYPTODIRA Cope, 1868
AMERICHELYDIA Joyce et al., 2013
CHELONIOIDEA Oppel, 1811
PAN-CHELONIIDAE Joyce, Parham & Gauthier, 2004
CTENOCHELYIDAE (new clade name)
Figs. 13–15

**Phylogenetic definition** ‘Ctenochelyidae’ refers to the clade arising from the most recent common ancestor of *Ctenochelys* (orig. *Toxochelys*) *stenoporus*, *P. matutina*, and *Peritresius* (orig. *Chelone*) *ornatus*. The term ‘Ctenochelyidae’ first appears in Karl, Biermann & Tichy (2012) but no explicit definition for the clade is provided. Based on the context in which the name is used, Karl, Biermann & Tichy (2012) appear to be using the term to refer to the clade formed by the recognized species of *Ctenochelys* however, this usage does not follow established taxonomic practices. The name Ctenochelyidae is herein reassigned to the phylogenetically defined clade formed by the species listed above in order to maintain nomenclatural consistency.

**Diagnosis** Members of Ctenochelyidae can be diagnosed relative to other turtles by the following combination of derived characters: the presence of an incipient secondary palate involving minor contributions from the palatine and vomer (ch. 40/1), a domed contribution of the anteroventral portion of the vomer to the palate roof (ch. 49/1), the presence of a rod-like rostrum basisphenoidale (ch. 86/1), posteromedial nuchal fontanelles (ch. 123/1), epineural ossifications (ch. 127/1), a high degree of shell and plastral fenestration (ch. 133/3), and a pronounced metischial process (ch. 234/1).

**Referred taxa** In addition to the species listed in the phylogenetic definition of the clade, Ctenochelyidae also includes *Ctenochelys acris*, *Prionochelys nauta*, *Peritresius martini* Gentry et al., 2018, and all of their descendants. It is possible that other Late Cretaceous durophagous stem cheloniids such as *Euclastes wielandi* (Hay, 1908) and *Pacifichelys urbanai* Parham & Pyenson, 2010 may be sister to Ctenochelyidae given that several cladistic studies of fossil marine turtles have placed various species of *Euclastes* and *Pacifichelys* on the stem of Cheloniidae (Lynch & Parham, 2003; Parham, 2005; Kear & Lee, 2006; Lapparent de Broin et al., 2014). However, due the limited postcranial material known for *Pacifichelys*, the complete absence of described postcranial material for *Euclastes,* and the tendency of species scored exclusively from cranial material to behave as rogue taxa in the present matrix (see Gentry et al., 2018), it is not possible to precisely determine their phylogenetic interrelationships.

**Remarks** Among Cretaceous pan-chelonioids, there appears to be a decrease through time in the size of the foramen palatinum posterius as well as an increase through time in the size of the plastral fontanelles, the size and anterior expansion of the medial processes of the pubes, and the degree to which the ventral most portion of the palatine and vomer contribute to the formation of a secondary palate (Fig. 14). This trend is exemplified by members of Ctenochelyidae and in *P. matutina*, there appears to have been a primitive undershelving of the choana formed by the palatines and vomer, similar to but not as complete as the condition observed in skulls assigned to more derived pan-cheloniids such as *Euclastes wielandi* (Hirayama & Tong, 2003; AMNH 30030, text-fig. 1, p. 847), *Pacifichelys hutchisoni* (Lynch & Parham, 2003; LACM 103351, fig. 2, p. 24), and *Allopleuron hofmanni* (Mulder, 2003; NHM 42913, pl. 11, p. 51). This durophagous feeding specialization of *Prionochelys* and at least one species of *Ctenochelys* (*C. acris*) may be one of the earliest examples of the evolution of this ecomorphology in total group chelonioids (Parham & Pyenson, 2010). Interestingly, *Toxochelys latiremis* is not recovered as a sister taxon to Ctenochelyidae nor is it recovered as a pan-cheloniid. Despite the occasional interpretation of *T. latiremis* as a stem cheloniid (Hirayama, 1997; Weems & Brown, 2017), *Toxochelys latiremis* is recovered here and more often as a stem chelonioid (Kear & Lee, 2006; Joyce, 2007; Sterli, 2010 (*Toxochelys latiremys* [sic]); Anquetin, 2012; Rabi et al., 2013 (*Toxochelys latiremys* [sic]); Cadena & Parham, 2015; Zhou & Rabi, 2015; Gentry, 2016; Gentry et al., 2018). The posteromedial nuchal fontanelles (ch. 123/1) and pronounced metischial processes (ch. 234/1) of the ctenochelyids (Fig. 15) are interpreted here as symplesiomorphies as both features are also found in the pan-chelonioid species *Toxochelys latiremis* (Nicholls, 1988; Matzke, 2009).

**Table:** 

CHELONIIDAE Bonaparte, 1832

Crown group cheloniids are united by a contact between the parietal and squamosal owing to a poorly developed temporal emargination (ch. 13/0), a loss of foramina praepalatinum on the ventral surface of the premaxillae (ch. 35/1), significant contributions to the upper triturating surface by the palatines (ch. 39/1), contact between the vomer and palatines anterior to the internal naris (ch. 41/1), a posteriorly expanded upper triturating surface which obscures the vomerine pillar in ventral view (ch. 50/2), a sizeable contribution to the triturating surface by the vomer (ch. 51/1), and the scar for the latissimus dorsi and teres major muscles being located at or near the middle of the humeral diaphysis (ch. 245/1). The loss of the foramen palatinum posterius was found to be a shared derived characteristic of crown cheloniids and *Dermochelys coriacea* (ch. 66/2).

## Discussion

### Epithecal ossifications in marine turtles

Although a number of fossil (*Calcarichelys gemma* Zangerl, 1953; *Allopleuron hofmanni*) and extant (juvenile *Caretta caretta*) marine adapted turtle possess keeled neurals, only members of Ctenochelyidae exhibit neural crests consisting of both thecal and epithecal ossifications (Fig. 13). These epithecal elements are positioned at or near the pinnacle of each neural crest and dorsal to the junction of the first and second suprapygal. *Prionochelys matutina* is unique in having a pair of epineurals at the peak of the second neural crest and a posteriorly oriented apex of the episuprapygal ossification. Aside from the Coniacian-Campanian genera *Ctenochelys* and *Prionochelys*, the Campanian-Maastrichtian taxon *Peritresius* also possessed epineurals (Baird, 1964; Gentry et al., 2018). Based on the presumed arrangement of the neurals, the epineurals of *Peritresius* are shifted posteriorly relative to the condition observed for *Prionochelys* and *Ctenochelys.*

The only other turtles known to possess any form of epithecal ossification are the dermochelyids though Cretaceous species often referred to the stem of Dermochelyidae (Protostegidae Cope, 1872) lack these elements entirely. Another Cretaceous species routinely assigned to the stem of *D. coriacea* is *Mesodermochelys undulatus* Hirayama & Chitoku, 1996 from the Maastrichtian of Japan. However, this species has more recently been shown to be a derived protostegid (Hirayama, 2007, preliminary result), a radiation of sea turtles that may or may not share a marine ancestor with chelonioids (Joyce, 2007; see discussion in Cadena & Parham, 2015). The Campanian species *Corsochelys haliniches* Zangerl, 1960 has also been described as a stem dermochelyid, however, the only unambiguously referred specimen of *C. haliniches* is the holotype, which possesses neither a neural series nor a complete skull and only a partial peripheral series and plastron. Tong & Hirayama (2004) described a partial hyoplastron from the Maastrichtian of Morocco as being similar to that of *C. haliniches* but given the partial nature of the specimen, conservatively referred it to Dermochelyidae gen. and sp. indeterminate. Based on the fact that *C. haliniches* is currently known from a single specimen which does not exhibit any of the diagnostic features of Dermochelyoidea, its placement as a stem dermochelyid is doubtful.

The earliest definitive dermochelyid known to possess the carapacial mosaic of epithecal osteoderms characteristic of *D. coriacea* is *Arabemys crassisculata* Tong et al., 1999 from the late Paleocene-early Eocene of Saudi Arabia (Joyce et al., 2013). The roughly 30 My temporal gap between the first occurrence of *Arabemys* and latest occurrence of *Ctenochelys* coupled with the presumably distant phylogenetic relationship and the presence of several possible stem dermochelyids lacking epithecal osteoderms makes it highly unlikely that the common ancestor of Ctenochelyidae, Dermochelyidae, and Cheloniidae possessed any form of developed epithecal element. It is far more likely that epithecal ossifications evolved independently on both the stem of Dermochelyidae and the stem of Cheloniidae. Unlike the Cenozoic cheloniids which subsequently lost epithecal ossifications, the epitheca of the dermochelyids continued to develop until the vast majority of the typical carapacial elements were lost (Versluys, 1914; Hay, 1922; Rieppel, 2013).

### Remarks on Late Cretaceous pan-chelonioid paleobiogeography

The regional endemism of many Late Cretaceous marine turtles is a well-documented paleobiographical phenomenon (Zangerl, 1953; Nicholls & Russell, 1990; Hirayama, 1997). It is possible that this perceived endemism may be the result of poorly defined autopomorphic character suites for Cretaceous pan-chelonioid species leading to either the misidentification of fossil material belonging to these species or the creation of morphologically ambiguous ‘junk’ taxa. However, with regard to North American lineages, it does appear that widely distributed taxa are somewhat less common than those currently thought to be regionally endemic (Gentry et al., 2018). Recently, studies of fossil pan-chelonioids from the U.S. Gulf Coastal Plain have identified material belonging to species historically thought to be endemic to the Western Interior Seaway (WIS) (*Toxochelys latiremis*—Gentry & Ebersole, 2018; *Ctenochelys stenoporus*—A Gentry, pers. obs., 2017) and others thought to have been restricted to the Northeastern Atlantic Coast (NEAC) (*Peritresius ornatus*—Gentry et al., 2018). The synonymy of *Prionochelys galeotergum* with *Prionochelys matutina* makes *P. matutina* one of only two species of Late Cretaceous pan-cheloniid identified from both the WIS and Mississippi Embayment (ME). Additionally, there is at least one report of possible *Prionochelys* material from the NEAC (Baird & Case, 1966). The discovery and description of more complete pan-cheloniid specimens from all three regions, especially those possessing shell-skull associations, will likely lead to the creation of more complete diagnostic character sets for these species and in turn, may lend additional support to the emerging pattern of shared pan-cheloniid taxa between the WIS, ME, and NEAC.

## Conclusions

Phylogenetic analysis places *Prionochelys matutina* on the stem of Cheloniidae as a member of a monophyletic grouping (Ctenochelyidae) with other North American Late Cretaceous pan-cheloniids, including *Ctenochelys stenoporus*, *Ctenochelys acris*, *Peritresius martini*, and *Peritresius ornatus*. The members of Ctenochelyidae possess incipient secondary palates, pronounced carapacial and plastral fontanelles at all stages of development, and are characterized by the presence of superficial ossifications at the apices of the neural keel elevations along the dorsal midline of the carapace. The epithecal osteoderms (epineurals) found in Ctenochelyidae are unique among turtles. The presence of epineurals in ctenochelyid turtles shows that epithecal ossifications arose independently in both leatherback (Dermochelyidae) and hard-shelled (Cheloniidae) marine turtles. Whether or not the epineurals of Ctenochelyidae are homologous with the dermal ossicles comprising the carapace of *Dermochelys coriacea* remains untested however, histological thin sectioning of dermochelyid and ctenochelyid epithecal elements may reveal meaningful information in future studies.

##  Supplemental Information

10.7717/peerj.5876/supp-1 Table S1 List of *Prionochelys* specimens examined for this study

10.7717/peerj.5876/supp-2 File S1 Scorings for the species included in the phylogenetic analysis

10.7717/peerj.5876/supp-3 File S2 Phylogenetic character list

10.7717/peerj.5876/supp-4 File S3 Molecular constraint tree

10.7717/peerj.5876/supp-5 Table S2 Measurements taken from select *Prionochelys* specimens

## Institutional abbreviations

ALMNH: Alabama Museum of Natural History, Tuscaloosa, USA
AMNH: American Museum of Natural History, New York City, NY, USA
FMNH: Field Museum of Natural History, Chicago, Illinois, USA
KUVP: University of Kansas Museum of Natural History, Lawrence, USA
LACM: Los Angeles County Museum of Natural History, Los Angeles, California, USA
MSC: McWane Science Center, Birmingham, AL, USA
NHM: Natural History Museum Maastricht, Maastricht, Netherlands
NJSM: New Jersey State Museum, Trenton, NJ, USA
ROM: Royal Ontario Museum, Toronto, Canada
USNM: United States National Museum, Washington D.C., USA
YPM: Yale Peabody Museum, New Haven, CT, USA

## Additional Information and Declarations

### Data Availability

The following information was supplied regarding data availability: Raw data is provided in the Supplemental Materials.
